# Understanding the transition from embryogenesis to seed filling in *Phaseolus vulgaris* L. non-endospermic seeds

**DOI:** 10.3389/fpls.2025.1597915

**Published:** 2025-05-21

**Authors:** Cláudia Lopes, Pedro Fevereiro, Susana de Sousa Araújo

**Affiliations:** ^1^ Plant Cell Biotechnology Laboratory, Green-it Research Unit, Instituto de Tecnologia Química e Biológica António Xavier, Universidade Nova de Lisboa (ITQB-NOVA), Oeiras, Portugal; ^2^ MORE - Laboratório Colaborativo Montanhas de Investigação, Edificio do Brigantia Ecopark, Bragança, Portugal; ^3^ CIMO, LA SusTEC, Instituto Politécnico de Bragança, Bragança, Portugal

**Keywords:** early seed filling, *Phaseolus vulgaris* L., seed histology, storage compounds, transcriptome

## Abstract

**Introduction:**

Common bean (*Phaseolus vulgaris* L.) is one of the most consumed grain legumes. These legumes are a major source of proteins and other important nutrients, especially in developing countries. Studying seed development in common bean is crucial for improving yield, nutrition, stress tolerance and disease resistance while promoting sustainable agriculture and food security, with its sequenced genome and available molecular tools making it an excellent research model. Despite advances in studying *P. vulgaris* seed development, the precise timing and molecular regulation of the transition from embryogenesis to seed filling remain poorly understood. Although *P. vulgaris* seeds at 10 days after anthesis (DAA) were previously characterized as being in the late embryogenesis stage, our previous studies suggested that this transition might occur earlier than 10 DAA, prompting us to investigate earlier developmental stages.

**Methods:**

To accomplish this goal, we conducted a comprehensive analysis at 6, 10, 14, 18 and 20 DAA, integrating morphological, histological, and transcriptomic approaches.

**Results and discussion:**

Morphological and histochemical data revealed that by 10 DAA, cotyledons are fully formed, but storage compound accumulation is only noticed at 14 DAA, indicating that the transition from embryogenesis to seed filling occurs between 10 and 14 DAA. Transcriptomic analysis further supported this finding, showing upregulation of genes associated with seed storage proteins, starch metabolism, and hormonal regulation at 14 and 18 DAA. This study redefines the developmental timeline of *P. vulgaris* seed filling initiation, bridging a critical knowledge gap in legume seed biology. Given the limited availability of histological studies on early *P. vulgaris* seed development, our findings provide essential insights into the structural and molecular events driving this transition. By refining the timing and regulatory mechanisms of early seed development, this study lays the groundwork for future research aimed at enhancing seed quality and resilience in legumes.

## 1 Introduction

The common bean (*Phaseolus vulgaris* L.) is one of the most consumed grain legumes worldwide. *P. vulgaris* represents an advantage in developing countries for their affordability and long storage life when compared to animal protein (De La Fuente et al., 2011; Castro-Guerrero et al., 2016). In Eastern and Southern Africa per capita consumption of common bean is around 40–50 kg per year and represents 50% of the grain legumes consumed worldwide (Ribeiro et al., 2012; Ghanbari et al., 2015). With the massive increase in human population, agriculture faces some challenges and it is estimated that a 30% increase in common bean yield is needed by 2050 (Porch et al., 2013). Seed yield is a multifaceted trait that represents the final outcome of an intricate growth and maturation process that is less known in non-endospermic seeds where endosperm is completely consumed during embryo development. Among the primary contributors to yield, seed dry weight plays a crucial role, thus modulating this trait can significantly influence overall seed yield (Li and Yang, 2014). *P. vulgaris* develops large and fleshy cotyledons to guarantee seed germination and plantlet development. Indeed, 90% of the total nutritive value of common bean seeds are in the cotyledons (Chávez-Mendoza et al., 2019). With the release of *P. vulgaris* genome sequence and other genetic tools (Collado et al., 2015; Schmutz et al., 2014), this species represents a suitable model for molecular studies aimed at improving and selecting desirable quality traits underlying the development of non-endospermic seed (Leitão et al., 2017; Leitão et al., 2021; Mendes et al., 2022).

Our team has been devoting major efforts in characterizing the molecular and metabolic processes underlying *P. vulgaris* non-endospermic seed development and maturation. In our previous work, we provide a comprehensive description of proteome dynamics in *P. vulgaris* seed development (Parreira et al., 2016). Through a high-throughput gel-free proteomics approach (LC-MS/MS), we analyzed seeds at 10, 20, 30 and 40 days after anthesis (DAA), defining the principal stages of *P. vulgaris* seed development and main metabolic pathways underlying these stages. At 10 DAA, seeds were identified as being at the late embryogenic stage, characterized by a high rate of cell division. By 20 DAA, seeds transitioned into the maturation/filling stage, marked by increased biomass accumulation driven by storage reserve synthesis. At 30 DAA, seed development reached the end of the filling stage, as biomass accumulation reached its peak, marking the onset of seed dehydration. Finally, by 40 DAA, seeds had undergone complete desiccation, finalizing their maturation process.

In a subsequent study, we provided new insights into DNA integrity maintenance during seed development, focusing on DNA damage repair and chromatin remodeling mechanisms that safeguard genome stability (Parreira et al., 2018). Using Massive Analysis of cDNA Ends (MACE) and digital PCR (dPCR), we conducted a qualitative analysis of gene expression dynamics across developmental stages, characterizing the molecular mechanisms governing this process. Our findings shed light on the critical role of DNA integrity maintenance in developing seeds, revealing how DNA repair pathways and chromatin remodeling contribute to genome stability throughout seed development.

In a follow-up study, we addressed a critical knowledge gap by elucidating the role of post-transcriptional regulation mediated by miRNAs in *P. vulgaris* seed development (Parreira et al., 2021). To achieve this, we employed a high-throughput non-coding transcriptomics approach, utilizing small RNA sequencing (sRNA-Seq) in combination with degradome analysis and target prediction algorithms to identify miRNAs and their respective targets. This analysis led to the identification of 72 known miRNAs and 39 novel miRNAs. Our findings revealed that miRNAs highly accumulated during early developmental stages play roles in regulating the end of embryogenesis, delaying the onset of seed maturation, and modulating storage compound synthesis and allocation. Notably, most miRNAs were most abundant at 10 DAA. Other studies have demonstrated that seed filling in Arabidopsis is primarily regulated by the LAFL network, which consists of the key transcription factors *LEC1, ABI3, FUS3*, and *LEC2* (Jo et al., 2019; Lepiniec et al., 2018; Boulard et al., 2017). In our previous work, we observed that at 10 DAA, *PHABULOSA (PHB)*, which promotes the seed maturation program by directly activating *LEC2* expression (Tang et al., 2012), was already present at relatively low levels, with only residual *LEC2* expression detected (Parreira et al., 2018). This suggests that the seed filling program activation might have occurred earlier than 10 DAA, the earliest time point analyzed by our previous LC-MS, MACE and sRNA-Seq.

This study addresses some key questions regarding the timing and molecular regulation of seed filling initiation in *P. vulgaris*. In view of our previous findings suggesting that the seed filling program can begin before the 10th DAA, the main objective was to test this hypothesis by determining the timing of this developmental transition and the molecular mechanisms underlying it. Specifically, we aimed to answer the following questions:

i) Does the transition to seed filling occur before 10 DAA?;
ii) At 10 DAA, is the seed still in the embryogenesis stage, or does it already have well-formed cotyledons?;
iii) What are the main molecular mechanisms and metabolic pathways regulating the transition from embryogenesis to seed filling?

To address these questions, we expanded our analysis to include earlier time points (6, 10, 14, 18 and 20 DAA) and employed a multidisciplinary approach that integrates morphological, histological and transcriptomic analyses. By refining the developmental timeline of seed filling initiation, this study provides a more detailed understanding of *P. vulgaris* non-endospermic seed development, contributing essential knowledge for crop improvement strategies.

## 2 Materials and methods

### 2.1 Plant material and growth conditions

In this study, the *Phaseolus vulgaris* genotype SER16, a red-seeded Mesoamerican variety provided by CIAT (International Center for Tropical Agriculture) was used. Recognized for its drought resistance and efficient remobilization of storage compounds to seeds, SER16 serves as an excellent model for research on seed development and stress adaptation (Polania et al., 2016). Additionally, it has been previously utilized in studies conducted by our laboratory (Parreira et al., 2016; 2018; 2021). Seeds were germinated onto water soaked paper in Petri dishes at 27°C for 2 days, followed by 3 days at 23°C, always in the dark as described in Parreira et al. (2016). Seedlings were individually transferred to 2.5 l pots containing a (2:1:1) mixture of commercial soil (Compo Sana S.A., Barcelona, Spain), peat and vermiculite, respectively. Seedlings were grown in growth chamber (Fitoclima 5.000 EH, ARALAB, Portugal) with controlled environmental conditions, with 50% humidity, photoperiod of 16/8-h day/night at 25/18°C, respectively and light intensity of 400 μmol m−2 s−1. Eighty plants were kept in the environmental conditions described above during the full experiment and watered 3 times per week.

*P. vulgaris* SER16 flowers were tagged and pods/seeds were harvested at 6, 10, 14, 18 and 20 days after anthesis (DAA). Harvested seeds were divided into 3 batches: one immediately frozen in liquid nitrogen and stored at −80°C for molecular (transcriptomic) analyses; another used to measure seed length, fresh and dry weight to characterize the seed development process, while the remaining batch was fixed for histochemical analyses.

### 2.2 Morphological characterization of the early steps of seed development

For the morphological analyses, 2 seeds coming from 15 individual plants were used in each of the studied time points (6, 10, 14, 18 and 20 DAA). Immediately after harvesting, seeds were photographed and seed length was measured, using ImageJ (Schneider et al., 2012). Then, those seeds were weighted to quantify the seed fresh weight. Later, after being submitted for 15 days to 70°C the seed dry weight was weighted.

### 2.3 Histology assays

For the histochemical analysis, 4 biological replicates (seeds) per time point (6, 10, 14, 18 and 20 DAA) were used. Seeds were submerged in ice-cold FAA fixative solution [FAA: 47.5% ethanol from Carlo Erba (Val de Reuil, France), 3.7% formaldehyde solution from Sigma-Aldrich (St. Louis, MO, USA) and 5% glacial acetic acid from Scharlau (Barcelona, Spain)]. The immersed material was exposed to moderate vacuum for 1 hour to pull the air out of the tissue and force the infiltration of the fixative solution. The material was fixed overnight at 4°C and then washed with TBS 1X (0.05M Tris-HCl from Carl Roth (Karlsruhe, Germany), 0.15M sodium chloride (NaCl) pH 7.6 from Merck (Darmstadt, Germany). Seeds were sectioned, by first securing the material in the vibratome’s block with a drop of Super Glue and set for 15–20 min at room temperature. Transversal sections at the seed hilum level with 25 µm were cut using the vibratome 1000 Plus. Each section was placed in a microscope slide coated with 200µl of poly(lysine) and left to dry. Six sections per fixed seed were stained with Calcofluor White Staining, Coomassie Blue Staining or Periodic acid Schiff to stain cellulose in cell walls, proteins and carbohydrates, respectively (Pellicciari and Biggiogera, 2017). Images from the sections were captured using a LEICA DM6 B microscope. Calcofluor White Staining images were captured using UV light, while for Coomassie Blue Staining and Periodic acid Schiff a bright-field lighting was used.

For each timepoint studied, Calcofluor slides were used to calculate cotyledon parenchyma cell area and cotyledon section area using ImageJ software (Schneider et al., 2012). To measure the cotyledon parenchyma cell area and cotyledon section area, the scale was first established, after which the cells and cotyledons were selected and analyzed using ImageJ. Coomassie and Periodic acid Schiff images were converted to a grayscale (16 bit), inverted and the mean gray value for pixel intensity retrieved by the software was used to estimate overall protein and carbohydrate accumulation.

### 2.4 RNA extraction, quantification and quality assessment

For total mRNA isolation, frozen seeds were ground to a fine powder in liquid nitrogen using a mortar and pestle. RNA was extracted as described in (Parreira et al., 2018). Traces of DNA contamination were removed with an Ambion^®^ TURBO™ DNase (Life Technologies, Carlsbad, CA, USA) following the manufacturer´s instructions. RNA quantification and purity were assessed using a NanoDrop™ 2000c Spectrophotometer (Thermo Fisher Scientific Inc., Waltham, MA, USA). Moreover, the Qubit^®^ 2.0 Fluorometer (Thermo Fisher Scientific Inc.) with RNA BR Assay Kit was used to quantify the RNA. RNA purity was estimated based on the A260/280 and A260/230 absorbance ratios and was approximately 2 before DNAse treatment. RNA integrity was assessed by electrophoresis in a 2.0% agarose gel, stained with SYBR^®^ Safe (Life Technologies). The absence of DNA contamination was verified by a standard polymerase chain reaction (PCR) using primers for the *P. vulgaris ACTIN* gene, gene ID: Phvul.001G142500 (**Supplementary Table 2**). RNA samples were stored at -80°C until needed.

### 2.5 RNA-sequencing

Twelve RNA-Seq libraries were constructed from three biological replicates from seeds harvested at 6, 10, 14 and 18 DAA. Due to the reduced size of the sampled seeds at 6 DAA, each biological replicate consists of a pool of 200 seeds randomly harvested from 80 plants. For seed samples harvested at 10, 14, 18 and 20 DAA, each biological replicate consists of a pool of 3 to 4 seeds harvested from the same individual. Libraries construction and sequencing was performed by NOVOGENE (Cambridge, UK). Pair-ended cDNA libraries were sequenced on Illumina NovaSeq 6000 platform following manufacturer’s recommendations and 150 bp paired-end reads were generated. mRNA was purified from total RNA using poly-T oligo attached magnetic beads and fragmented randomly in fragmentation buffer [NEBNext First Strand Synthesis Reaction Buffer (5X)], followed by cDNA synthesis using random hexamers and reverse transcriptase (RNase H-). Second‐strand cDNA synthesis was subsequently performed using buffer (Illumina) with dNTPs, RNase H and *Escherichia coli* polymerase I to generate the second strand by nick-translation. The final cDNA library is ready after a round of purification, adenylation of 3′ ends of DNA fragments, A-tailing, ligation of sequencing adapters, size selection of cDNA fragments of preferentially 150 bp in length with ligated adaptor molecules on both ends were selectively enriched using Illumina PCR Primer Cocktail in a 10 cycle PCR. Library concentration was quantified using a Qubit 2.0 fluorometer (Life Technologies). Insert size was checked on an Agilent 2100 and quantified using qPCR.

### 2.6 RNA-seq bioinformatic analysis

The original raw data from Illumina are transformed to Sequenced Reads by base calling. Raw data are recorded in a FASTQ file. Raw reads are filtered to remove reads with adapter contamination or reads with low quality. Only clean reads were used in the downstream analyses. The percentage of bases whose correct base recognition rates are greater than 99% and 99.9% (Q20, Q30, respectively), GC content and sequence duplication level of the clean data were calculated. All the downstream analyses were based on the clean data with high quality. Pair-ended clean reads were mapped with HISAT2 (HISAT version 2.1.0; Kim et al., 2019) to the reference genome (*P.vulgaris* v2.1, U.S. Department of Energy Joint Genome Institute, Phytozome v12.0: http://phytozome.jgi.doe.gov/). HISAT2 is a fast and sensitive alignment program for mapping next-generation sequencing reads, enabled effective alignment of RNA-seq reads, particularly, reads spanning multiple exons. Because transcriptome annotation is still incomplete, this RNA-seq study revealed novel genes and transcripts. To do that, Cufflinks Reference Annotation Based Transcript (RABT) assembly method was used to assemble the set of transcript isoforms of each bam file obtained in the mapping step. This was done using ‘Cuffcompare’ that compares Cufflinks assemblies to reference annotation files and help sort out new genes from known ones.

Genes were considered expressed if they present an average read raw number ≥ 100 at least in one of the studied timepoints. The expected number of Fragments Per Kilobase of transcript sequence per Millions base pairs sequenced (FPKM) which takes into account the effects of both sequencing depth and gene length on counting of fragments was also used to establish gene expression abundances and profiles using HTSeq software (Anders et al., 2014). Differential expression analysis was performed using the DESeq2 R package (1.12.0; TNLIST, Beijing, China). Pair-wise comparisons between two consecutive timepoints (6 vs 10 DAA, 10 vs 14 DAA and 14 vs 18 DAA) were established. A Benjamini-Hochberg correction, for estimating false discovery rates (FDR) was also applied to the analysis made (Benjamini and Hochberg, 1995). Genes which presented a corrected p‐value ≤ 0.001 and |Log2(Fold Change - FC) | ≥1 were considered differentially expressed genes (DEGs).

Functional characterization was performed using the MapMan web tools (http://www.plabipd.de/portal/mercator-sequence-annotation). Protein sequences were obtained using BioMart (*Phaseolus vulgaris* genome version 2.1) in Phytozome v.12 (https://phytozome.jgi.doe.gov/) to create a mapping file for the Mercator pipeline. Some genes were also annotated using Phytozome v.12 BLAST.

With the differential expression analysis data, Venn diagrams were generated and obtained from the Venny 2.1 (Oliveros, 2015) and used to infer the overall distribution of differentially expressed genes (DEGs). Heatmaps were constructed using Morpheus online tool, (https://software.broadinstitute.org/morpheus), to show the normalized gene expression among the samples. Clustering was performed based on Euclidean distance and average linkage.

### 2.7 RT-qPCR validation of RNA-seq data

Expression of 15 selected genes were analyzed by RT-qPCR (**Supplementary Table 2**) on a Light Cycler^®^ 480 System, using the LightCycler^®^ 480 SYBR Green I Master protocol. These genes were chosen to represent a broad range of differential expression and total transcript counts. One μg of total RNA from 3 biological replicates per time point (6, 10, 14 and 18) was reverse transcribed, using the High-Capacity cDNA Reverse Transcription Kit (Applied Biosystems, Foster City, CA, USA) following the manufacturer’s instructions. Primers were designed using the Primer3 software and primer sequences are listed in **Supplementary Table 2**. PCR amplification efficiencies were tested for all primers for target and reference genes using cDNA two-fold dilution series. Using the geNorm and NormFinder software packages from the GenEx v.5 software (MultiD, Goteborg, Sweden), two reference genes: *EUKARYOTIC RELEASE FACTOR 1 (eRF1)* family protein (*PEL1*; Phvul.010G077790.1) and *RING/U-BOX SUPERFAMILY PROTEIN* (*XERICO*; Phvul.008G190100.1) used by Parreira et al. (2021), were selected for the gene relative expression analysis.

Thermo cycling reactions were carried out following the described conditions: denaturation step at 90°C for 5 min; 45 cycles of amplification at 95°C for 10 s; 10 s at 60°C and 10 s at 72°C. For each reaction, a melting curve (dissociation stage) was performed to detect non-specific PCR products and/or contaminants. A non-template control (NTC), without cDNA, was also included for each primer mix to detect possible contaminations.

### 2.8 Data availability

RNA-seq data were deposited in the NCBI Sequence Read Archive BioProject under the accession number PRJNA1224322 (SRR32361921, SRR32361920, SRR32361917, SRR32361916, SRR32361915, SRR32361914, SRR32361913, SRR32361912, SRR32361911, SRR32361910, SRR32361919 and SRR32361918).

### 2.9 Statistical analyses

Data from the morphological analysis (seed length, fresh and dry weight) and from histochemical analysis (mean gray value for pixel intensity in Coomassie and Periodic acid Schiff images) was analyzed with IBM SPSS Statistics V25.0 software. Data statistical significance was assessed using one-way analysis of variance (ANOVA) coupled with *post-hoc* Tukey HSD for mean pairwise comparison. Means were considered significantly different when P ≤ 0.05.

## 3 Results

### 3.1 In *P. vulgaris* SER16 seeds, the accumulation of reserves starts to occur between 10 and 14 DAA

**Figure 1:**
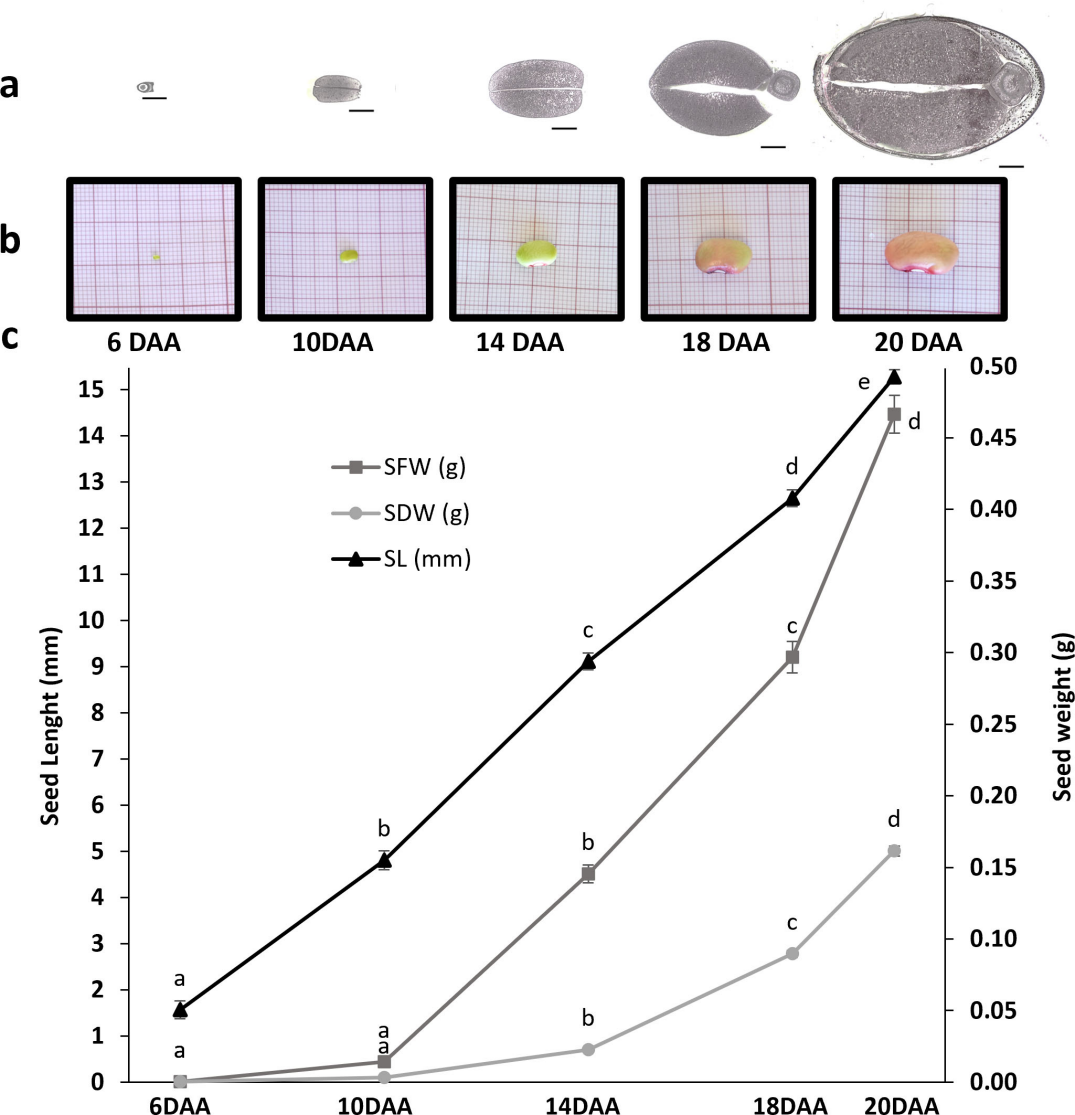
Characterization of seed development in *P. vulgaris* SER16 at 6, 10, 14, 18 and 20 DAA (days after anthesis). The study was focused on the transition from seed embryogenesis to seed filling. **(a)** transversal section of seeds at 6, 10, 14, 18 and 20 DAA at the Hilum level, black scale bars indicate 1 mm; **(b)** Photographs of seeds at 6, 10, 14, 18 and 20 DAA; **(c)** Seed fresh weight (SFW), Seed dry weight (SDW), Seed length (SL). Error bars represent the standard deviation and different letters indicate statistically significant differences between time points (p<0.05).

A significant increase in seed length was observed throughout all the time points studied (p-value < 0.05) (**Figure 1**). No significant differences were observed in the seed fresh weight (SFW) and seed dry weight (SDW) between 6 and 10 DAA samples, although a slight increase in SFW is observed. However, between 10 and 14 DAA a significant increase, with a Fold change (FC) of 8.49 in the SFW and a FC of 7.27 in SDW were observed, suggesting that the accumulation of reserves starts to occur around 10DAA. Between 14 and 18 DAA, there was still a significant increase in SFW (FC=2.41) and SDW (FC= 3.76). Between 18 and 20 DAA, the increase was less prominent with a FC of 1.88 in the SFW and a FC of 1.85 in SDW. **Figure 1** illustrates a noticeable increase in seed size over time. Additionally, the same figure highlights a gradual change in seed coat color transitioning from green to orange until 20 DAA.

**Figure 2:**
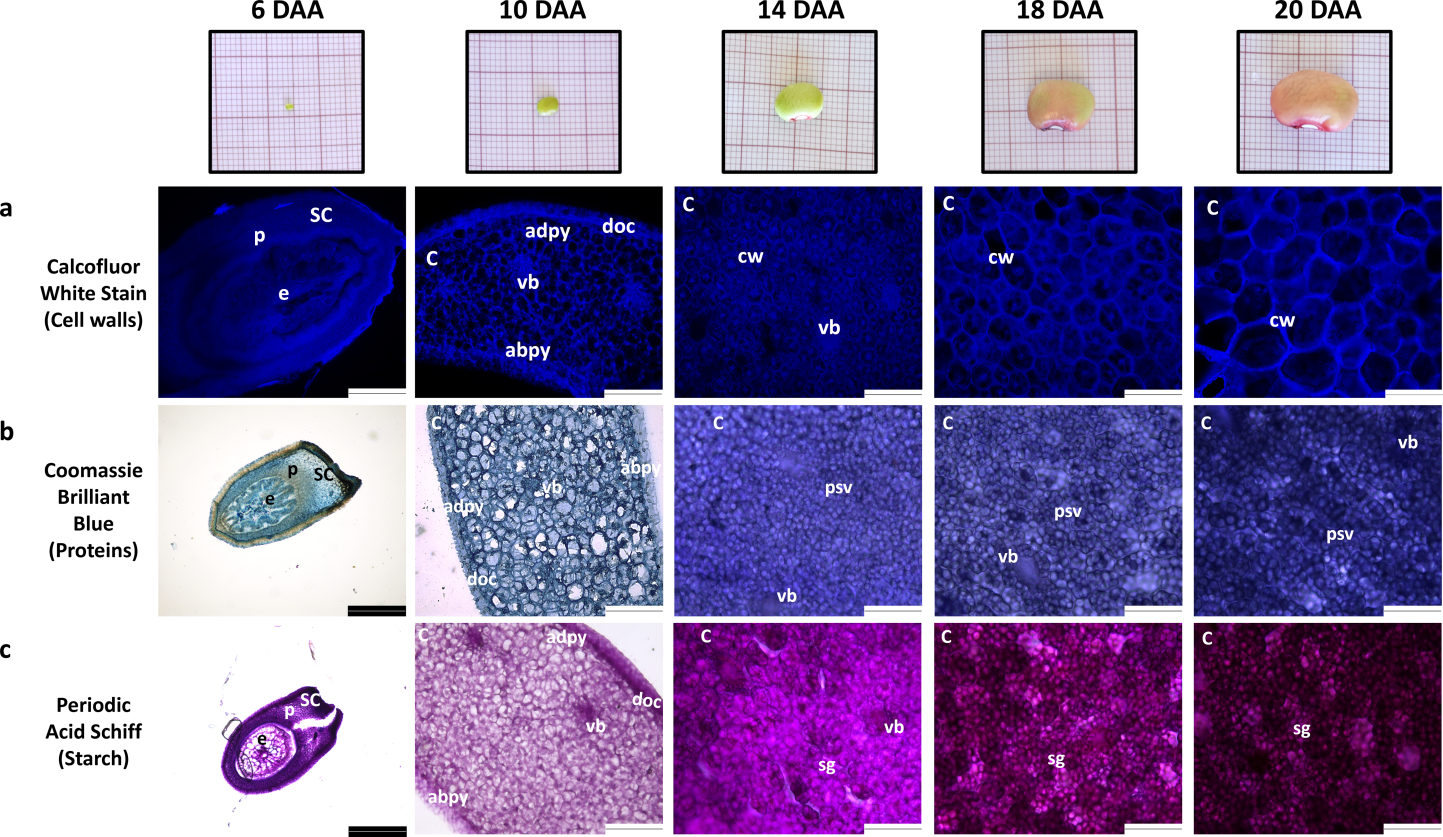
Morphology and histology of developing *P. vulgaris* SER16 seeds harvested at 6, 10, 14, 18 and 20 days after anthesis (DAA). Histological observations of: **(a)** cell walls, **(b)** protein and **(c)** starch accumulation during seed development. The cell walls, proteins and starch were stained with Calcofluor white stain, Coomassie Brilliant Blue and Periodic Acid Schiff, respectively. The white scale bars indicate 124.4 µm and black scale bars indicate 248.9µm; abpy - cotyledon abaxial parenchyma; adpy - cotyledon adaxial parenchyma; C, cotyledon; cw, cell wall; doc, dermal cell complex; e, embryo; p, parenchyma; psv, protein storage vacuoles; SC, seed coat; sg, starch grains; vb, vascular bundle.

At 6 DAA the seeds were still under embryogenesis stage since no clear differentiation of cotyledons from the embryo is noticed (**Figure 2**). At 10 DAA, the cotyledons were clearly developed, in which it was possible to observe cotyledon parenchyma cells, dermal cell complex and vascular bundles (**Figure 2**). Furthermore, at 10 DAA, the visible stained structures are the cell walls, as detected by Coomassie Blue and Periodic Acid-Schiff staining.

By 14 DAA, protein storage vacuoles and starch grains are already visible and are also stained, indicating that starch and storage protein accumulation begins between 10 and 14 DAA (**Figures 2b, c**), suggesting that the transition from embryogenesis to seed filling occurs between 10 and 14 DAA. Due to the staining of protein storage vacuoles and starch grains with Coomassie Blue and Periodic Acid-Schiff, cell walls cannot be easily distinguished in these images at these time points.

**Figure 3:**
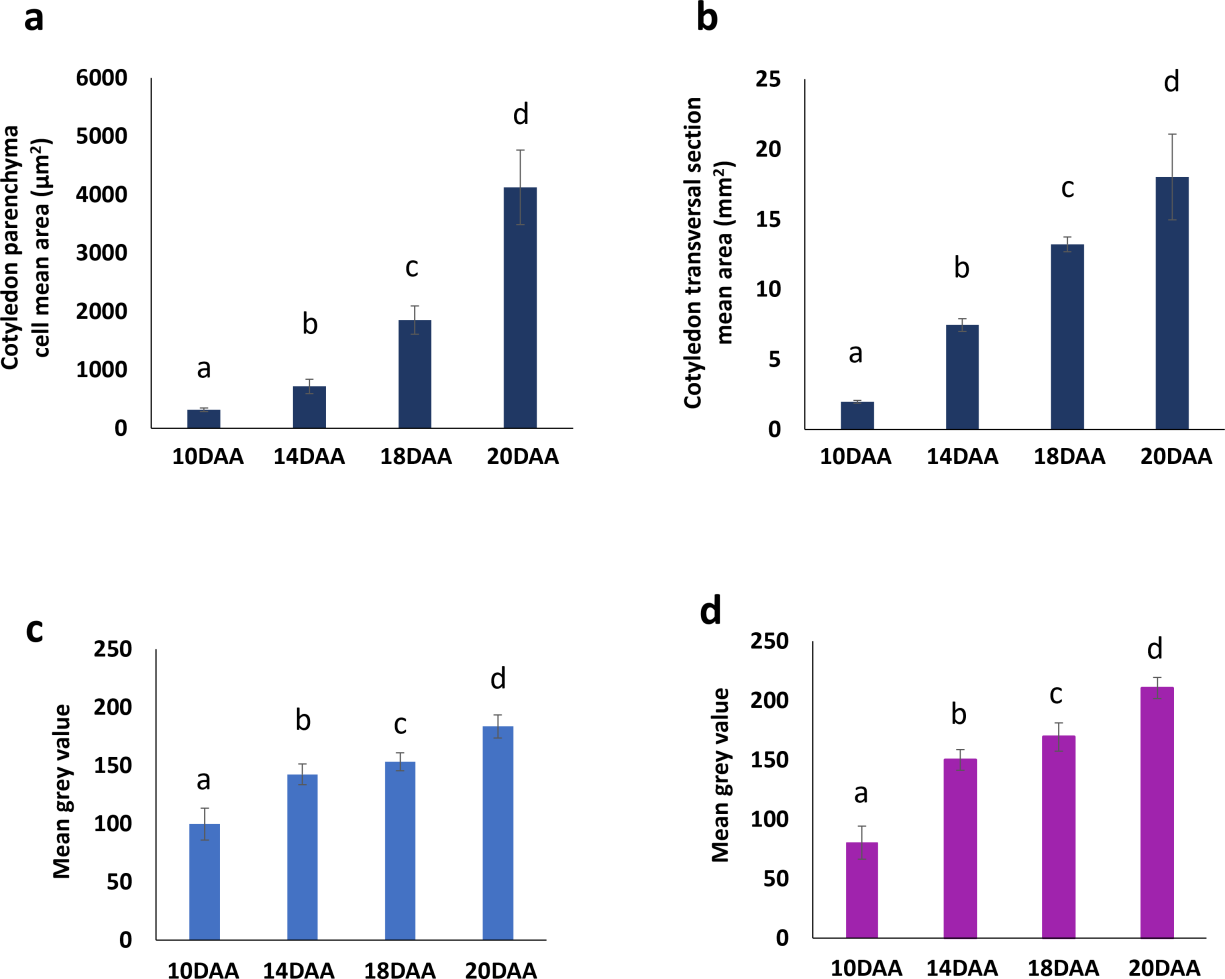
Changes in cotyledon parenchyma cell number and area of *P. vulgaris* SER16 seeds, from 10 to 20 DAA (days after anthesis). **(a)** Cotyledon parenchyma cell mean area. **(b)** Cotyledon transversal section mean area. Mean gray value for pixel intensity (obtained using Image J software after converted to a grayscale) for: **(c)** Coomassie blue and **(d)** Periodic acid Schiff. Different letters above each bar indicates statistically significant differences (p < 0.05).

Coomassie Blue and Periodic Acid-Schiff images were converted to a grayscale and the mean gray value for pixel intensity was used to estimate overall protein and starch accumulation. Values from 10 DAA correspond to cell walls staining, since no protein storage vacuoles or starch grains were visible (**Figures 2b, c**). Protein and starch accumulation increased significantly from 14 DAA to 18 DAA, followed by a more pronounced rise from 18 DAA to 20 DAA (**Figures 3c, d**). This trend is supported by the mean gray value FC measurements: Coomassie Blue staining shows a FC= 1.08 from 14 to 18 DAA and a FC=1.19 from 18 to 20 DAA. Moreover, Periodic Acid-Schiff staining shows a FC= 1.13 from 14 to 18 DAA and a FC=1.24 from 18 to 20 DAA (**Figures 3c, d**).

The cotyledon section area, at the hilum level, increased approximately four-fold (FC=3.98) from 10 to 14 DAA and almost doubled (FC=1.70) from 14 to 18 DAA (**Figures 1b**, **3b**). Cotyledon parenchyma cells approximately doubled their area along the time points studied (10 vs 14 FC=2.19; 14 vs 18 FC=2.79; 18 vs 20 FC=2.39) (**Figure 3a**). These results suggest that from 10 to 14 DAA cells stopped to divide and start to expand to accumulate storage compounds.

### 3.2 Global overview of the gene expression from late embryogenesis to seed filling

The RNA-Seq of 12 libraries from three biological replicates from seeds harvested at 6, 10, 14 and 18 DAA, produced 884.1 million 150-bp reads, with an average of 68 millions of mapped reads/sample (**Table 1**). A total of 17191 genes, with ≥ 100 average raw reads at least in one studied timepoint, were considered expressed in this study (**Supplementary Table 1**). Sequences annotated as *BOWMAN-BIRK SERINE PROTEASE INHIBITOR FAMILY* (Phvul.004G134000; Phvul.004G133900), *CONCANAVALIN A-LIKE LECTIN PROTEIN KINASE FAMILY PROTEIN* (Phvul.004G158200), *LECTIN RECEPTOR KINASE A4.3* (Phvul.004G158100) and *RMLC-LIKE CUPINS SUPERFAMILY PROTEIN* (Phvul.007G192800) were among those with highest total raw read counts, suggesting no ribosomal RNA (rRNA) contamination during library preparation.

**Table 1:** Characterization of the 12 RNA-seq libraries generated for developing seeds of *P. vulgaris* SER16.

Sample	Number of raw reads	GC (%)	Q20(%)	Q30(%)	Number of mapped reads	Mapped reads (%)
*SD6DAA1*	35262765	45.77	97.47	92.73	34733045	98.5
*SD6DAA2*	31498837	44.67	97.21	92.27	30689952	97.4
*SD6DAA3*	39111219	44.99	97.50	92.78	38286149	97.9
*SD10DAA1*	43099217	45.91	97.55	92.88	42453531	98.5
*SD10DAA2*	39512074	46.76	97.52	92.84	38753821	98.1
*SD10DAA3*	50089372	46.43	97.58	92.98	49139349	98.1
*SD14DAA1*	35309229	46.23	97.64	93.08	34644029	98.1
*SD14DAA2*	37942658	46.13	97.73	93.29	37228676	98.1
*SD14DAA3*	37260953	46.25	97.72	93.24	36568854	98.1
*SD18DAA1*	33694888	46.01	97.48	92.77	33030091	98.0
*SD18DAA2*	34481992	46.51	97.59	93.01	33905182	98.3
*SD18DAA3*	33312508	45.34	97.52	92.88	32615875	97.9

**Figure 4:**
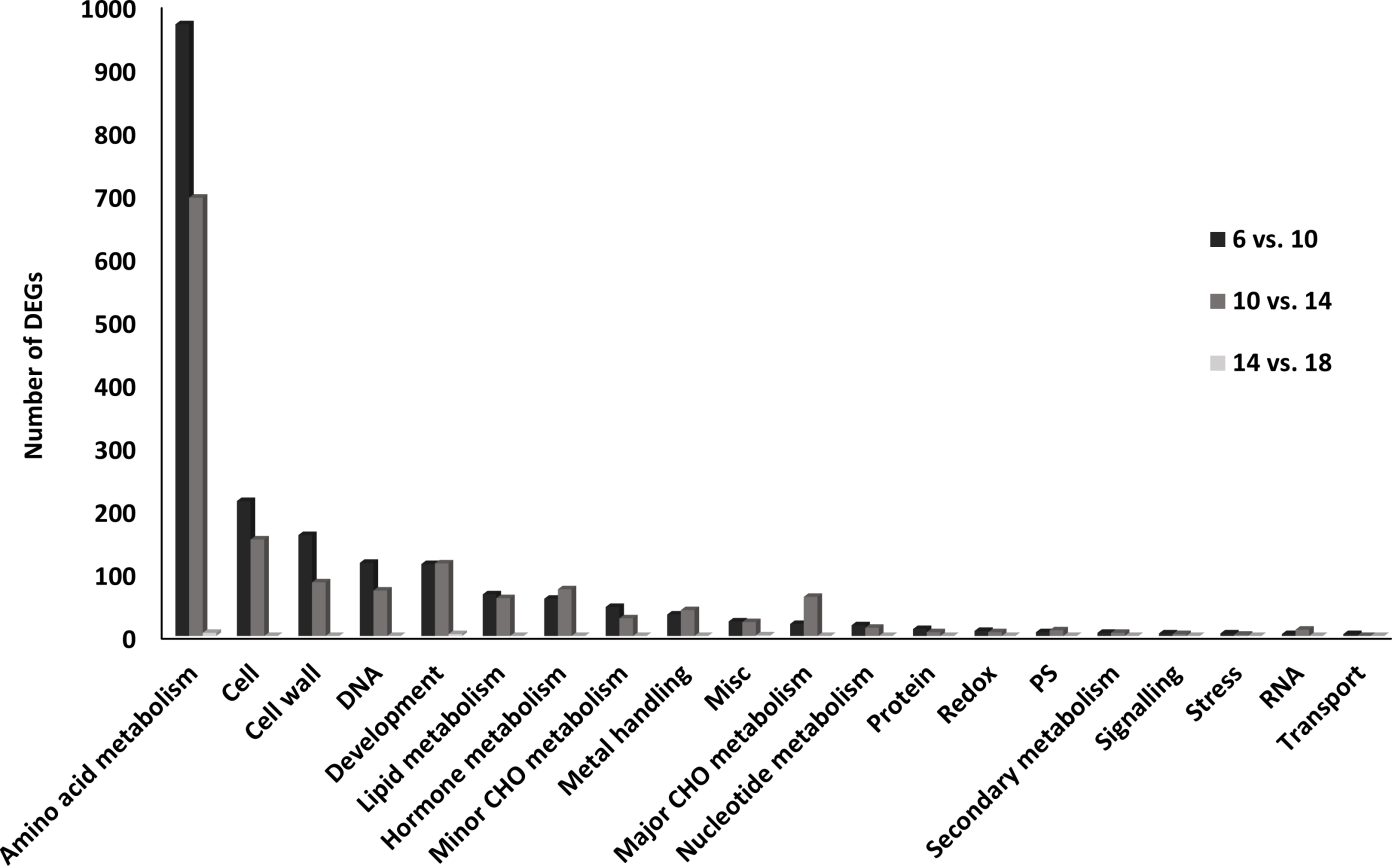
Functional categories of differentially expressed genes (DEGs) in the transition from the embryogenesis to seed filling in *P. vulgaris* SER16 seeds. The percentage of DEGs in each category were displayed between the main comparison studied (6 DAA vs 10 DAA; 10 DAA vs 14 DAA and 14 DAA vs 18 DAA). The percentage of DEGs changed was calculated by comparison of the number of DEGs in each category in relation to the total of DEGs identified within each comparison.

The most represented functional category was “Amino acid metabolism” [MapMan BINCODE (BC 13), followed by “Cell” (BC 31), “Cell wall” (BC 10), “DNA” (BC 28), “Development” (BC 33), “Lipid metabolism” (BC 11), “Hormone metabolism” (BC 17) and “Minor CHO metabolism” (BC 3) (**Figure 4**).

### 3.3 Expression of genes involved in seed storage protein accumulation and carbohydrate synthesis

**Figure 5:**
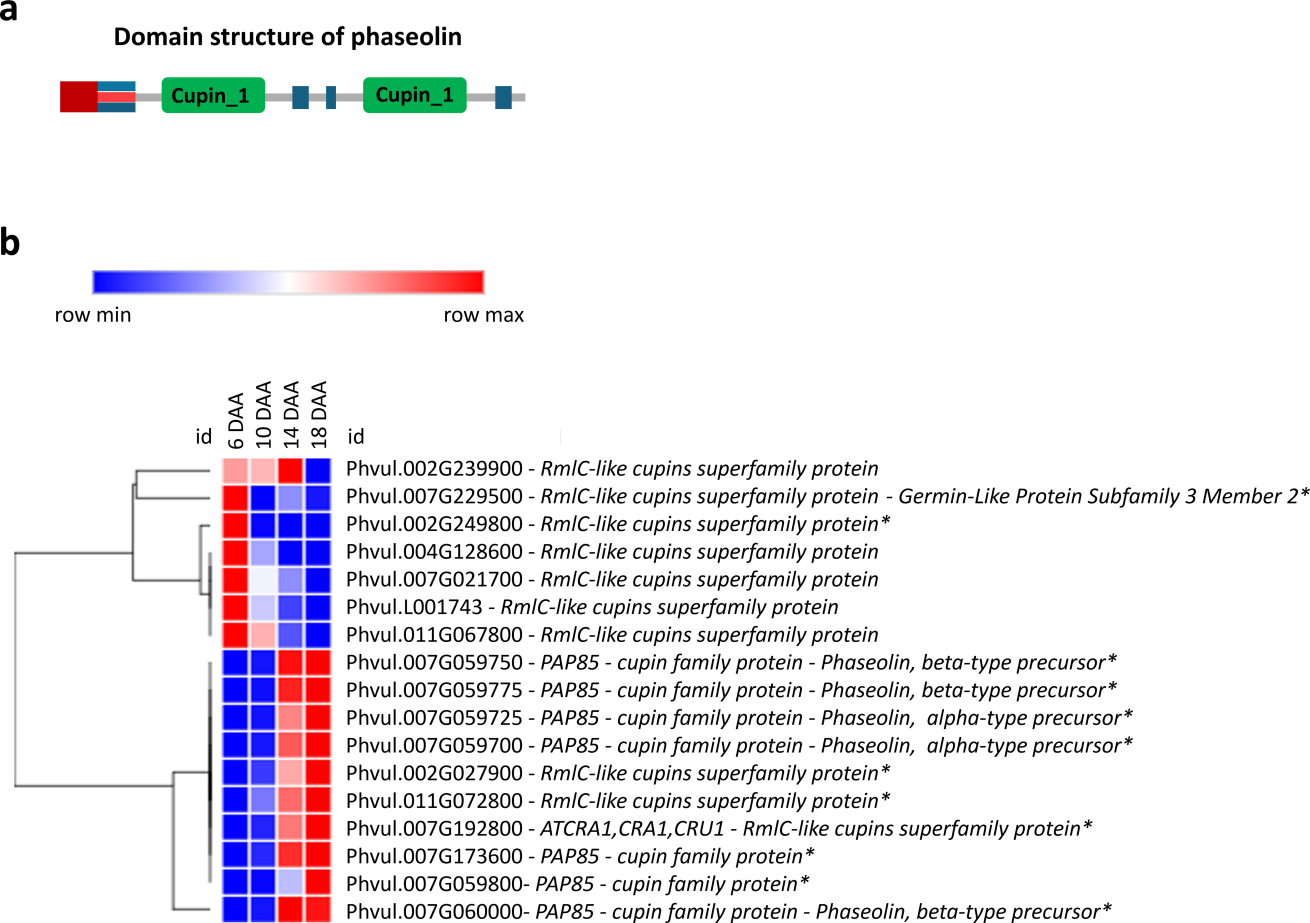
Seed storage protein gene expression. **(a)** Domain structure of Phaseolin exhibiting the bi-cupin architecture (Emani and Hall, 2008); **(b)** Heatmap of transcripts related to seed storage proteins at 6, 10, 14 and 18 days after anthesis (DAA). FPKM were clustered using Euclidean distance and an average linkage. Blue color represents low expression levels (lower FPKM values); White (Middle Color) represents moderate expression levels and Red represents high expression levels (higher FPKM values). Asterisks indicate differentially expressed genes (DEGs): corrected p‐value ≤ 0.001 and |Log2(Fold Change - FC)≥1.

Transcripts annotated as belonging to the Cupin superfamily, some of whose genes encode the phaseolin storage protein, were analyzed (**Figure 5**). A cluster of genes of this family is upregulated at 6 DAA and a second cluster is upregulated at 14 and 18 DAA. Furthermore, most of the amino acid activation, protein synthesis, protein folding and protein post-translational modification genes are upregulated at 6 and 10 DAA, with a smaller cluster upregulated at 14 and 18 DAA (**Supplementary Figures 1**-**4**).

**Figure 6:**
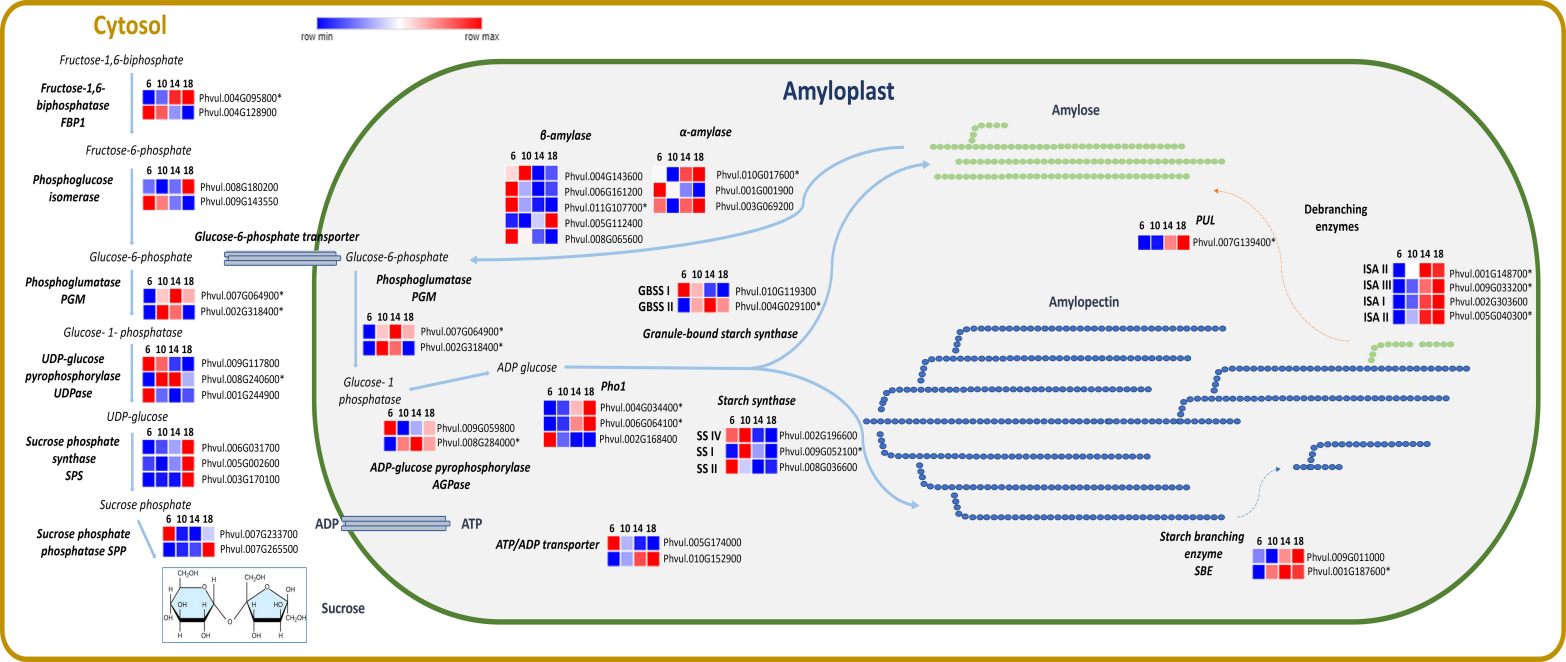
Heatmaps of transcripts of *P. vulgaris* SER16 seeds, categorized in MapMan, related to sucrose and starch synthesis at 6, 10, 14 and 18 days after anthesis (DAA). *Fructose 1–6 biphosphatase (FBP1), Phosphoglucose isomerase, Phosphoglumatase (PGM), Sucrose phosphate synthase (SPS), Sucrose phosphate phosphatase (SPP), UDP-glucose pyrophosphorylase (UDP), ADP-glucose pyrophosphorylase (AGPase), Plastid starch phosphorylase (Pho1), Starch synthase (SS), Granule-bound starch synthase (GBSS), Starch branching enzyme (SBE), Isoamylases (ISA), Pullulanase (PUL), α-amylase and β-amylase*. Schematic representation of sucrose and starch synthesis adapted from (Qu et al., 2018). FPKM were clustered using Euclidean distance and an average linkage. Blue color represents low expression levels (lower FPKM values); White (Middle Color) represents moderate expression levels and Red represents high expression levels (higher FPKM values). Asterisks indicate differentially expressed genes (DEGs): corrected p‐value ≤ 0.001 and |Log2(Fold Change - FC)≥1.

The expression of transcripts categorized in MapMan related to genes involved in the sucrose and starch synthesis (**Figure 6**) was analyzed. Among those, *PHOSPHOGLUMATASE (PGM)* (EC. 5.4.2.2) was more expressed after 10 and 14 DAA. Additionally, *SUCROSE PHOSPHATE SYNTHASE (SPS)* (E.C. 2.4.1.14) showed an upregulation at 18 DAA. Starch molecular structure is composed by two polysaccharides: amylose and amylopectin. *GRANULE-BOUND STARCH SYNTHASE (GBSS)* (E.C. 2.4.1.242) is consistently upregulated across all time points. The gene of the *1,4-ALPHA-GLUCAN BRANCHING ENZYME (SBE)* (E.E. 2.4.1.18) that catalysis the branched structure of the amylopectin, was upregulated after 10 DAA. The gene of *ISOAMYLASES (ISO)* (E.C. 3.2.1.68) essential for amylopectin crystallization, exhibited increased expression at 14 and 18 DAA. The upregulation of *SBE* and *ISO* genes at 14 and 18 DAA, which contribute to amylopectin formation, suggests enhanced carbohydrate accumulation at this stage of seed development.

### 3.4 Expression of genes involved in the LAFL regulatory network

**Figure 7:**
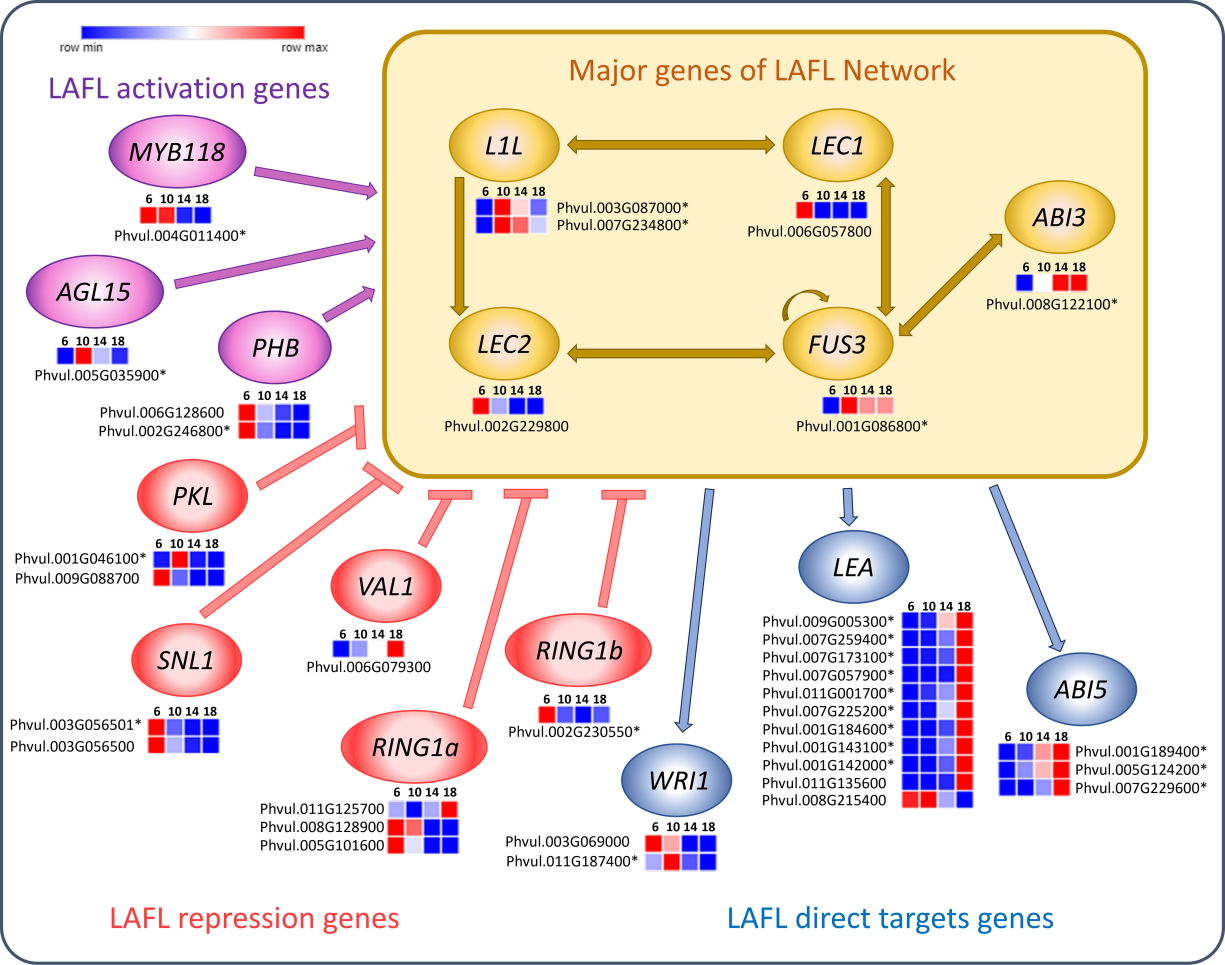
Heatmaps of transcripts of *P. vulgaris* SER16 seeds, categorized in MapMan, related to LAFL network genes found differentially expressed between 6, 10, 14 and 18 days after anthesis (DAA). LAFL activation genes are shown in purple, LAFL repression genes in pink and LAFL direct target genes in blue. The yellow box highlight the major genes of the LAFL network (*LEC1, L1L, ABI3, FUS3* and *LEC2*) and illustrates their regulatory interactions. Schematic representation adapted from (Devic and Roscoe, 2016; Jia et al., 2014). FPKM were clustered using Euclidean distance and an average linkage. Blue color represents low expression levels (lower FPKM values); White (Middle Color) represents moderate expression levels and Red represents high expression levels (higher FPKM values). Asterisks indicate differentially expressed genes (DEGs): corrected p‐value ≤ 0.001 and |Log2(Fold Change - FC)≥1.

Seed development is regulated by the LAFL transcription factors network, which consists of the key genes *ABI3 (Abscisic Acid Insensitive 3), FUS3 (FUSCA3), LEC1 ABI3 (ABSISIC ACID INSENSITIVE 3), FUS3 (FUSCA3), LEC1 (LEAFY COTYLEDON 1), LEC2 (LEAFY COTYLEDON 2) and L1L (LEC1-LIKE)* (**Figure 7**). Among these, LEC1 and LEC2 were upregulated at 6 DAA and *FUS3* and *ABI3* were upregulated at 10, 14 and 18 DAA. Moreover, *ABI5 (ABA INSENSITIVE 5)* showed increased expression at 14 and 18 DAA. *LEA (LATE-EMBRYOGENESIS- ABUNDANT)* was predominantly upregulated at 18 DAA, with a smaller cluster exhibiting upregulation at 6 and 10 DAA.

**Figure 8:**
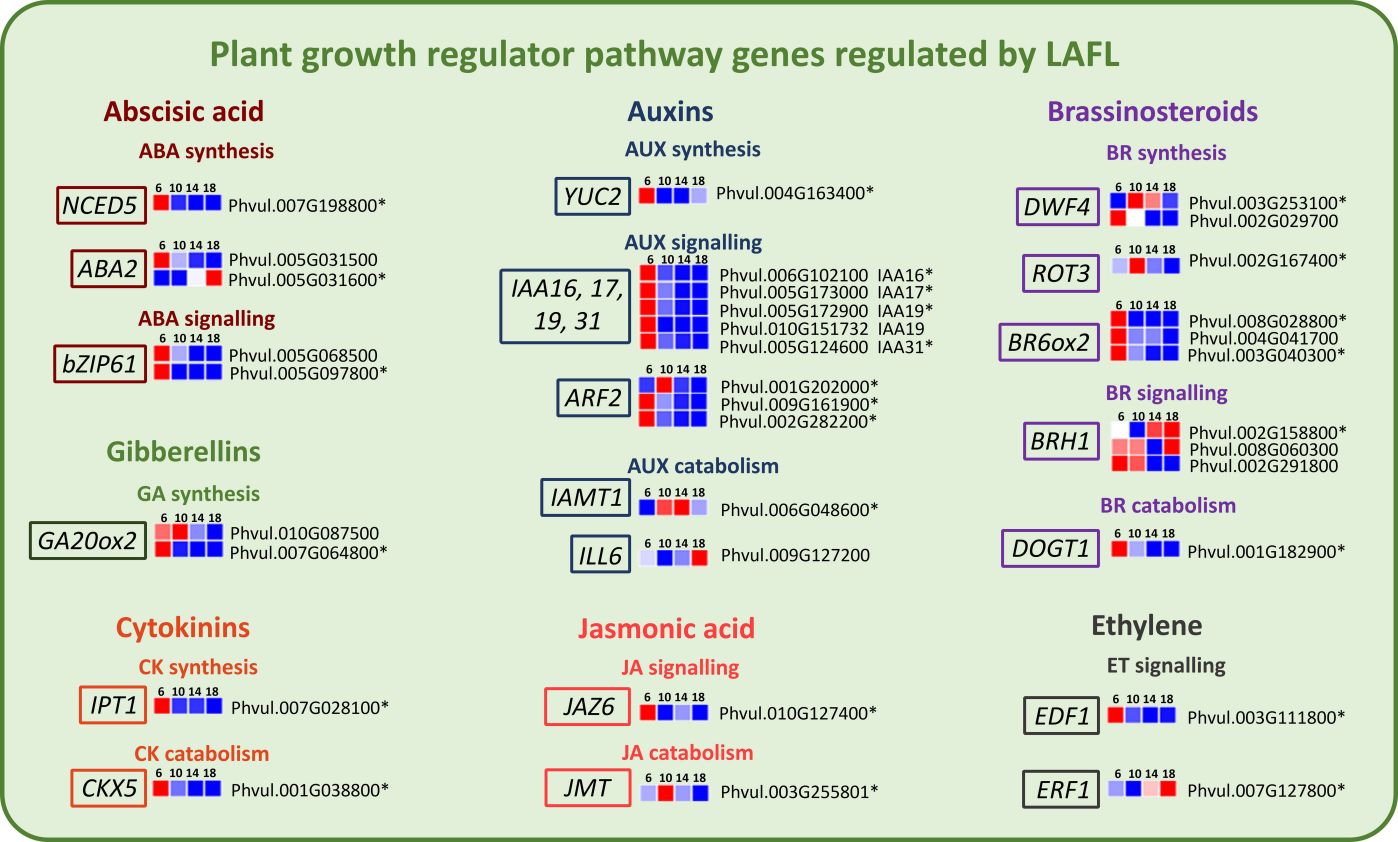
Heatmaps of transcripts of *P. vulgaris* SER16 seeds, categorized in MapMan, related to plant growth regulator pathways, including key components of the abscisic acid (ABA), jasmonic acid (JA), cytokinin (CK), gibberellin (GA), ethylene (ET), auxin (AUX) and brassinosteroid (BR) pathways, which are regulated by the LAFL network, found differentially expressed between 6, 10, 14 and 18 days after anthesis (DAA). FPKM were clustered using Euclidean distance and an average linkage. Blue color represents low expression levels (lower FPKM values); White (Middle Color) represents moderate expression levels and Red represents high expression levels (higher FPKM values). Asterisks indicate differentially expressed genes (DEGs): p‐value ≤ 0.001 and |Log2(Fold Change - FC)≥1.

Genes involved in the metabolism of plant growth regulators, modulated by LAFL Network proteins were also found expressed among the analyzed time points (**Figure 8**). In general, most of these genes are upregulated at 6 DAA. Exceptions are one of the ABA2 genes, involved in ABA synthesis, the *IAMT1* gene involved in Auxin catabolism, one of the *DWF4* genes, involved in Brassinsteroid (BR) synthesis, two of the *BRH1* genes involved in BR signaling and *ERF1* a key regulator in Ethylene signaling. These genes were upregulated at 14 or 18 DAA.

Genes associated with cell division showed an upregulation at the first time points studied (6 and 10 DAA) of seed development (**Supplementary Figure 5**). A smaller subset of these genes exhibited sustained expression at later stages. Furthermore, genes related to cell wall expansion were identified from RNA-Seq data. Those genes showed an upregulation at the first time points studied (6 and 10 DAA) of seed development (**Supplementary Figure 6**). A smaller group of these genes continued to be expressed during the later stages. Genes related to cell wall synthesis showed an upregulation throughout all the time points studied of seed development (**Supplementary Figure 7**).

### 3.5 Validation of RNA sequencing data by RT-qPCR

A strong positive correlation (0.79 ≤ R² ≤ 1.00) was observed between RNA-Seq expression values (FPKM) and the corresponding average RT-qPCR expression levels (relative units) for each of the fifteen selected genes, measured at 6, 10, 14 and 18 DAA (**Supplementary Table 3**).

## 4 Discussion

Seed development in *P. vulgaris* SER16 follows a well-orchestrated series of events that include embryogenesis, reserve accumulation and maturation. Our previous studies on seed development from 10 to 40 DAA established a comprehensive molecular and proteomic landscape underlying later stages of development, including the transition from storage compound deposition to desiccation (Parreira et al., 2016; 2018; 2021). However, unresolved questions remained: What are the histological changes underlying the SD stages studied? When the transition from embryogenesis to seed filling does occur? What are the main molecular mechanisms and metabolic pathways regulating the transition from embryogenesis to seed filling? To address this, we expanded our analysis to earlier time points (6, 10, 14, 18 and 20 DAA) using a multidisciplinary approach, integrating morphological, histological and transcriptomic analyses.

### 4.1 Morphological, histochemical and transcriptomic analysis shows that the transition from late embryogenesis to seed filling begins around 10 DAA

Morphological measures revealed a continuous increase in seed length from 6 to 20 DAA. While seed fresh weight (SFW) and seed dry weight (SDW) exhibited a slight increase from 6 to 10 DAA (**Figure 1**), a pronounced accumulation was observed from 10 DAA onwards, suggesting a shift toward storage reserve deposition. Histochemical analysis reinforces this developmental progression. As shown in **Figure 2**, embryogenesis is still in progress at 6 DAA. At 10 DAA, the cotyledons are fully formed, but protein granules are not seen yet, indicating that the transition to seed filling occurs only after 10 DAA.

Moreover, the observed increase in cell area and cotyledon section size between 10 and 14 DAA (**Figures 3a, b**) suggests a transition from active cell division to cell expansion, a hallmark of seed filling where cells allocate space for reserve deposition. The observed upregulation of genes related to cell division and expansion at 6 and 10 DAA (**Supplementary Figures 5**, **6**), alongside continuous activation of cell wall synthesis genes (**Supplementary Figure 7**), aligns with the histological results, reinforcing the hypothesis that the transition from embryogenesis to seed filling is marked by a shift from cell proliferation to expansion.

A similar growth regulation pattern has been described in other studies in chickpea, where early embryogenesis is primarily driven by cell division (Turner et al., 2005; Weber et al., 2005), followed by cotyledon differentiation, which coincides with sucrose uptake, cell expansion, and the accumulation of storage compounds (Dante et al., 2014). According to Coelho and Benedito (2008), the embryogenesis phase in *P. vulgaris* is characterized by cell division and differentiation, which contribute to the formation of the embryo tissues and the endosperm. The rapid increase in SFW relative to SDW during this phase, observed in our study, further suggests an influx of water that facilitates cell expansion, a process commonly observed during early seed filling stages (Bewley et al., 2013).

### 4.2 Seed storage reserve accumulation occurs between 10 and 14 DAA

The expression of genes involved in the synthesis and accumulation of seed reserves follows the morphological and histochemical findings. The upregulation of RmlC-like cupins superfamily genes at 6 DAA (**Figure 5**) may be related to their dual role in embryogenesis and stress response, as some early-expressed storage proteins contribute to protecting the developing embryo (Neutelings et al., 1998; Dunwell et al., 2000). Germin-like proteins were previously identified as a highly homogeneous group of proteins with oxalate oxidase activity and they were found expressed in the embryo axis of *P. vulgaris* during germination (Aubry et al., 2003). Notably, *GERMIN-LIKE PROTEIN SUBFAMILY 3 MEMBER 2*, expressed at 6 DAA, has been associated with somatic and zygotic embryogenesis and proposed to play a role in desiccation/hydration processes and oxidative stress protection (Neutelings et al., 1998; Khuri et al., 2001). Additionally, GERMIN-LIKE PROTEINS exhibit superoxide dismutase (SOD) activity, which mitigates oxidative stress during early seed development, ensuring proper cellular function and metabolic stability (Khuri et al., 2001). The early activation of these protective mechanisms suggests that seed development at 6 DAA is already metabolically active, preparing for reserve accumulation and structural modifications essential for seed filling.

However, genes that encode PHASEOLIN, a major *P. vulgaris* storage protein, start to increase their expression at 10 DAA and are all highly upregulated at 18 DAA, suggesting that the seeds are starting the seed filling stage at 10 DAA, which correlates with the histochemical detection of storage compounds and the significant increase in seed dry weight observed during this period. Such results are also aligned with our previous studies (Parreira et al., 2016), where we found that seed storage proteins as as LEGUMIN (UniProKB ID: F8QXP7), PHASEOLIN (UniProKB ID: Q43632) and ALPHAPHASEOLIN (UniProKB ID: X5CHW7 and X5D3J1) showed a significant accumulation from 10 to 20 DAA.

The upregulation, at 6 DAA of genes involved in protein amino acid activation, synthesis, folding, and post-translational modification (**Supplementary Figures 1**-**4**) suggests that seeds are already preparing for reserve synthesis and accumulation at that stage. Likewise, Parreira et al. (2016) observed at 10 DAA, a high accumulation of proteins associated with protein synthesis (e.g 40S RIBOSOMAL PROTEIN S2-4 (UniProKB ID: V7C8K4 (A0A0B2QJI4)) and 60S RIBOSOMAL PROTEIN L6 (UniProKB ID: V7C7H0 (A0A0B2SQP6)), folding (e.g CHAPERONE PROTEIN ClpC, CHLOROPLASTIC (UniProKB ID: V7AZ24 (A0A0B2PK21)), targeting and post-translational modification (e.g UBIQUITIN CARBOXYLTERMINAL HYDROLASE (UniProKB ID: V7ASH7)), in early seed development. The early activation of these processes is crucial, as seed storage proteins must undergo proper folding and post-translational modifications to ensure functional stability and efficient accumulation during seed filling. This aligns with previous studies showing that post-translational modifications, such as glycosylation and disulfide bond formation, play essential roles in stabilizing storage proteins and optimizing their deposition within protein bodies (Önder et al., 2022). Our findings indicate that even though storage protein accumulation does not begin until 10 DAA, the molecular machinery required for their synthesis is already being established at 6 DAA.

### 4.3 Starch accumulation starts after 10 DAA and show a complex interplay among different starch biosynthetic enzymes

*P. vulgaris* seeds have one of the highest percentage of amylose in their starch grains. Amylose contents from 32.40% to 49.73% were reported by Du et al. (2014), while Bajaj et al. (2018) found an amylose content of 49.50% in *P. vulgaris*. The histochemical analysis show that starch starts to accumulate after 10 DAA (**Figure 2**). The continuous increase in transcription observed for one of the two *ATP/ADP* transporter genes (**Figure 6**), which is the more highly expressed isoform, particularly from 10 DAA onward, appears to be consistent with this observation. Previous studies showed that starch accumulation in potato tubers is strongly influenced by alterations in plastidic *ATP/ADP* transporter activity (Tjaden et al., 1998).

In our study, a higher *GBSSI* expression at 6 DAA was observed, followed by a decline until 18 DAA. Despite the increase of *GBSSII* until 14 DAA, it is possible that additional enzymatic or regulatory mechanisms may contribute to amylose biosynthesis. The anticipation of the expression of the *GBSSI* may be linked to the fact that it may also participate in amylopectin biosynthesis (Pfister and Zeeman, 2016). It was shown that *GBSS* enzymes are involved in amylopectin synthesis by extending its side chains and aiding in the formation of elongated glucan structures (Hanashiro et al., 2008). Similar patterns have been observed in *Brachypodium distachyon*, where *GBSSI* is highly expressed in immature seeds but decreases in later stages of development (Chen et al., 2014). Moreover, our previous proteome study showed high levels of *ISA3* protein (Parreira et al., 2016), along with the increased transcript levels of *ISA3* and *PUL* gene expression between 10 and 20 DAA (Parreira et al., 2018), which further supports the hypothesis that starch debranching enzymes contribute to amylose accumulation. This suggests that *GBSS* activity alone may not fully account for amylose accumulation, and other enzymatic pathways, to be elucidated, may play a role in the storage of high amylose percentage in *P. vulgaris*.

In *P. vulgaris* seeds Takashima et al. (2007) showed that PvISAI/II have high activity for amylopectin, whereas PvISAIII shows a preference for dextrin and glycogen over amylopectin and has high specificity for short-chain removal (DP 2 to 4) (Takashima et al., 2007). Although the role of *ISAIII* in starch biosynthesis remains uncertain, its highest expression at 14 and 18 DAA in our data suggests a potential involvement in amylose synthesis. We propose that *ISAIII* facilitates this process by modifying the starch granule surface, likely enhancing the availability of elongated glucan chains for GBSS-independent amylose-like molecules formation during these stages. This is further supported by the increased expression of *PULLULANASE (PUL)* at 14 and 18 DAA, which has been identified as a key enzyme in amylopectin remodeling through debranching, leading to increased amylose content as part of a cold-adaptive response (Thakur et al., 2021).

These findings suggest the interplay between *GBSS, SBE, SS, ISA* and *PUL* enzymes may compensate for the decline in *GBSS* expression from 6 to 18 DAA, ensuring continued amylose accumulation in *P. vulgaris* seeds. It is possible that this corresponds to a different pattern in starch assemblage in relation to other seeds.

### 4.4 LAFL network regulation in *P. vulgaris* SER16: LEC1/LEC2 initiate embryogenesis, FUS3/ABI3 drive seed filling, and LEA/ABI5 regulate desiccation

The LAFL transcription factors modulate embryo development and maturation (Lepiniec et al., 2018). Our results show an early upregulation of *LEC1* and *LEC2* at 6 DAA (**Figure 7**), which is aligned with *LEC1* being a central regulator of seed development, controlling embryo morphogenesis, photosynthesis and seed maturation (Jo et al., 2019). In soybean, *GmLEC2a* expression was found to be highest during the early stages of seed development, followed by a decline as the seeds matured (Manan et al., 2017). Furthermore, Manan et al. (2017) also demonstrated that *LEC2* plays a role in regulating carbon partitioning, influencing the synthesis of triacylglycerol, carbohydrates and proteins in soybean. Moreover, in our previous work, we observed that *PHABULOSA (PHB)* and *LEC2* were already present at 10 DAA at relatively low levels (Parreira et al., 2018), suggesting that the peak of LAFL activation occurs before 10 DAA. Indeed, we found MIR166 members could play a role in tuning the levels of several HD-ZIP transcription factors including *PHB* in *P. vulgaris* SER16 developing seeds (Parreira et al., 2021). The higher expression of *LEC1* and *LEC2* at 6 DAA supports this finding, indicating that the molecular framework for seed filling is already in place before 10 DAA, even though the histological changes associated with reserve accumulation occur after this date.

Expression of *FUS3* and *AB13* starts to increase at 10 DAA (**Figure 7**) correlating with the onset of seed filling, coinciding with the starting of the significant increase in SFW and SDW (**Figure 1**). This finding aligns with *FUS3* playing a crucial role in the acquisition of embryo-dependent seed dormancy, the determination of cotyledonary cell identity, and the regulation of storage compound synthesis and accumulation (Tiedemann et al., 2008). *ABI3* loss-of-function in *Medicago* mutants show impaired expression of storage protein genes, *LEA* genes, secondary metabolism genes, and cell cycle-related genes throughout seed maturation (Lalanne et al., 2021). Additionally, in *Linum usitatissimum*, overexpression of LuABI3–1 or LuABI3–2 significantly increased seed fatty acid and storage proteins (Liu et al., 2023), reinforcing *ABI3*’s role in controlling reserve deposition during seed maturation.

Furthermore, we observed an upregulation of *LEA* and *ABI5* at 14 and 18 DAA (**Figure 7**), which suggests that desiccation-related mechanisms are progressively activated alongside reserve accumulation, rather than being restricted to the final maturation phase. It was demonstrated in soybean that *LEA* proteins help protect and stabilize desiccation-sensitive proteins and plasma membranes during periods of dehydration (Shih et al., 2010). Notably, *LEA* proteins in soybeans were found to be more highly expressed in fully matured dry seeds, reinforcing their potential role in preserving cellular structures during desiccation (Jones and Vodkin, 2013). Furthermore, research in *P. vulgaris* has demonstrated that the PvLEA-18 protein responds to dehydration and accumulates in the seed during the final stage of seed development (Colmenero-Flores et al., 1999). The increased expression of ABI5 may be linked to its role as a key regulator of late seed maturation in legumes. This is supported by findings in *Pisum sativum* and *Medicago truncatula*, where *abi5* knockout mutants exhibited severe impairments in both seed longevity and dormancy acquisition (Zinsmeister et al., 2016). Additionally, Zinsmeister et al. (2016) demonstrated that *ABI5* regulates *LEA* protein expression, as *abi5* mutants showed a significant reduction in *LEA* proteins. Two new miRNAs, miR_6 and miR_18 targeting *LEAs*, *EM1* and *RAB18* transcripts respectively, were found highly expressed in 5 and 10 DAA, decreasing afterwards in developing *P. vulgaris* SER16 seeds (Parreira et al., 2021). On the same work, opposite profiles were shown for *EM1* and *RAB18* transcripts expression and protein accumulation. These results reinforce the functional role of *LEA* proteins in safeguarding cellular integrity during quiescence and acquisition of desiccation tolerance. Additionally, these findings indicate that seed mechanisms related to desiccation are progressively activated in *P. vulgaris* mid-filling, rather than being restricted to the final maturation phase. Additionally, post-transcriptional regulatory mechanisms mediated by miRNAs play role in tuning these responses.

### 4.5 LAFL-regulated phytohormones orchestrate early seed development and maturation in *P. vulgaris* SER16

A key function of the LAFL network is re-programming of the major plant hormone signaling pathways in the seed (Jia et al., 2014). Our results show that cytokinin, gibberellin, and auxin pathways are actively involved in early seed development, setting the molecular framework necessary for the later transition into seed filling. The upregulation of *IPT1* (biosynthesis) and *CKX5* (catabolism) at 6 DAA (**Figure 8**) implies a fine-tuned balance between cytokinin accumulation and degradation, likely contributing to early embryogenesis progression. In another grain legume, pea, an increase in cytokinin levels in the seed coat of transgenic peas was associated with higher expression of cell wall invertase (*CWIN*) in the seed coat and correlated with an increase in sucrose levels in the cotyledon, influencing nutrient allocation and storage compound accumulation in seeds (Grant et al., 2021). This agrees with our findings in *P. vulgaris* SER16, where the early expression of cytokinin-related genes at 6 DAA likely contributes to the metabolic activity required to establish source-sink relationships before seed filling begins.

The upregulation of *GA20ox2* (gibberellin biosynthesis) at 6 and 10 DAA (**Figure 8**) suggests that gibberellins facilitate early cell expansion and cotyledon differentiation, preparing the seed for subsequent storage compound deposition. In a previous study, *GA 20-OXIDASE* genes from French bean were expressed in young seeds, similar to pea, where their highest expression was observed in very young seeds (until 4 DAA) (García-Martínez et al., 1997). Furthermore, in pea seeds, *PsGA20ox* genes were high in the embryo during early seed development, demonstrating that GA_1_ plays a key role in stimulating rapid branched parenchyma cell expansion. As the seed starts to mature GA levels start to decrease to limit embryo axis growth and allowing embryo maturation to proceed (Nadeau et al., 2011). The presence of *GA20ox2* expression at 10 DAA aligns with histological evidence showing that cotyledons are fully developed at this stage, yet storage compounds have not yet begun to accumulate. This supports the idea that early *GA20ox2* upregulation is essential for initial seed expansion and differentiation, reinforcing the developmental shift from active growth to storage phase initiation.

The upregulation of *IAA16, IAA17, IAA19, IAA31*, and *YUC2* (key auxin biosynthesis genes) at 6 DAA (**Figure 8**) indicates an active auxin signaling network governing early development. Auxins play a crucial role in embryo patterning, determining the apical-basal axis and promoting cell division and elongation (Smit and Weijers, 2015). This aligns with findings in *Phaseolus coccineus*, where the highest concentration of total IAA was detected in early-heart stage embryos, highlighting the importance of auxins in early seed development (Picciarelli et al., 2001). Similarly, in pea, auxins are essential for normal seed size and starch accumulation (McAdam et al., 2017). This is particularly relevant when considering that there were no significant differences observed between 6 and 10 DAA in SFW and SDW, reinforcing the idea that hormonal regulation at this stage is primarily preparing the seed for later expansion and reserve accumulation.

The upregulation of *NCED5* (a key enzyme in ABA biosynthesis) and *bZIP61* (a regulator of ABA signaling) at 6 DAA (**Figure 8**) aligns with the early activation of cell division genes and precedes the upregulation of genes associated with storage compound biosynthesis at later stages. In line with statement, we found that in SER16 developing seed, some members of the MIR167 potentially repress *NCED1* expression likely contributing to tune ABA levels during *P. vulgaris* seed development (Parreira et al., 2021). These finding support that in *P. vulgaris* SER16, ABA plays an early role in seed maturation initiation and in preparation for seed filling. In soybeans, ABA enhances carbon allocation and partitioning to the seeds (Reinoso et al., 2011). In pea seeds, the SnRK1 kinase was shown to interact with the ABA signaling pathway, serving as a crucial regulator in developmental programming and having a key role in orchestrating the transition from the pre-storage phase to maturation, ensuring proper seed development (Radchuk et al., 2006).

The upregulation of *ERF1* (a key regulator in ethylene signaling) and *BRH1* (a brassinosteroid-responsive *RING-H2* gene) at 14 and 18 DAA (**Figure 8**) suggests that, in *P. vulgaris* SER16, ethylene and brassinosteroids contribute to the regulation of storage compound accumulation and seed maturation. Brassinosteroids have been shown to enhance grain filling in rice by stimulating the flow of assimilates from source to sink, thereby increasing seed weight and overall yield (Wu et al., 2008). Moreover, in pea seeds, the amount of endogenous active Brassinosteroids increased when seeds were rapidly growing (during seed filling) but dropped significantly once the seeds were fully expanded and green (Nomura et al., 2007). This pattern is consistent with the role of brassinosteroids in promoting growth and development during early seed expansion. Moreover, in support of this, transcriptomic data from our previous study (Parreira et al., 2018) showed that key brassinosteroid-related genes, including *ROT3* and *BR6ox2*, are highly expressed at 10 DAA, with a gradual decline observed at later stages, further suggesting that brassinosteroids play a prominent role during early seed development. These findings support the idea that brassinosteroids regulate seed development through mechanisms that affect both embryo growth and nutrient mobilization, which likely contributes to the sustained increase in seed size and storage compound accumulation observed in *P. vulgaris* SER16.

## 5 Conclusion

This study provides the first comprehensive transcriptomic dataset, morphological and histolochemical data spanning from 6 to 18 DAA during the early stages of *Phaseolus vulgaris* seed development. These results provide a high-resolution perspective on the intricate and molecular mechanisms governing the transition from late embryogenesis to seed filling. Our findings indicate that while changes related to reserve accumulation occur after 10 DAA, the molecular framework for seed filling is established earlier, starting at the late embryogenesis. Likewise, the preparation for seed desiccation is established during the filling stage. The integration with the results obtained with previous works done in this experimental system (Parreira et al., 2016, 2018, 2021) provided biological evidence that an intricate and complex regulatory network is governing the seed filling mechanisms.

Preparation to seed filling, happening between 6 and 10 DAA, is marked by the early establishment of the molecular framework required for this process. The transcription factors *LEC1* and *LEC2* were upregulated at 6 DAA, along with cytokinin-related genes, suggesting early regulatory activation. Auxin biosynthesis and ABA-related genes were also more expressed at 6 DAA, likely influencing cell division, elongation before the onset of seed filling. The expression of *GA20ox2* at this stage indicates a transition from active growth to storage phase initiation. Additionally, the upregulation of genes involved in protein synthesis, folding, and modification at this stage further supports early molecular preparation for reserve accumulation.

Seed filling stage is characterized by protein and starch accumulation, which begins around 10 DAA. The expression of *FUS3* and *ABI3* starts to increase at 10 DAA, correlating with the onset of seed filling and coinciding with a significant rise in SFW and SDW. At this stage it is clear the increase of the expression of phaseolin genes, which guarantees the accumulation of this reserve protein. In relation to starch accumulation, high synthesis of amylose is supported by the interplay among *GBSS, SBE, SS, ISA* and *PUL* enzymes which compensate for the decline in *GBSS* expression from 6 to 18 DAA. In particular, there was high expression of *ISA* III and *PUL* at 14 and 18 DAA, probably involved in modifying amylopectin and facilitating the formation of GBSS-independent amylose molecules.

During mid-filling, between 14 and 18 DAA, desiccation tolerance genes, including *LEA* proteins and *ABI5*, are progressively activated, rather than being restricted to the final maturation phase. At this stage the upregulation of *ERF1* and *BRH1* at 14 and 18 DAA contributes to the regulation of storage compound accumulation and seed maturation.

These insights significantly advance our understanding of the regulatory networks involved in seed development and lay the groundwork for future efforts to optimize storage compound accumulation in legumes. By identifying early activated transcription factors and metabolic pathways, this study highlights valuable molecular targets for breeding programs aimed at enhancing seed quality traits such as protein content and starch composition. Still functional validation studies are needed to corroborate suggested roles played by selected genes and underlying mechanisms in *P. vulgaris* developing seeds. Furthermore, the molecular framework established here offers a valuable reference for cross-species comparisons in non-endospermic legumes, enabling translational research toward the development of resilient, nutrient-rich legume cultivars that support sustainable agricultural practices.

## Data Availability

RNA-seq data were deposited in the NCBI Sequence Read Archive BioProject under the accession number PRJNA1224322 (SRR32361921, SRR32361920, SRR32361917, SRR32361916, SRR32361915, SRR32361914, SRR32361913, SRR32361912, SRR32361911, SRR32361910, SRR32361919 and SRR32361918).
