# Supplementary material for: Understanding the transition from embryogenesis to seed filling in Phaseolus vulgaris L. non-endospermic seeds
Source: Front Plant Sci. 2025 May 21;16:1597915. doi: 10.3389/fpls.2025.1597915 (PMC12133513; doi:10.3389/fpls.2025.1597915)
Supplement: Supplementary file 1 [file DataSheet1.docx]

Supplementary Material

# Supplementary Figures

**
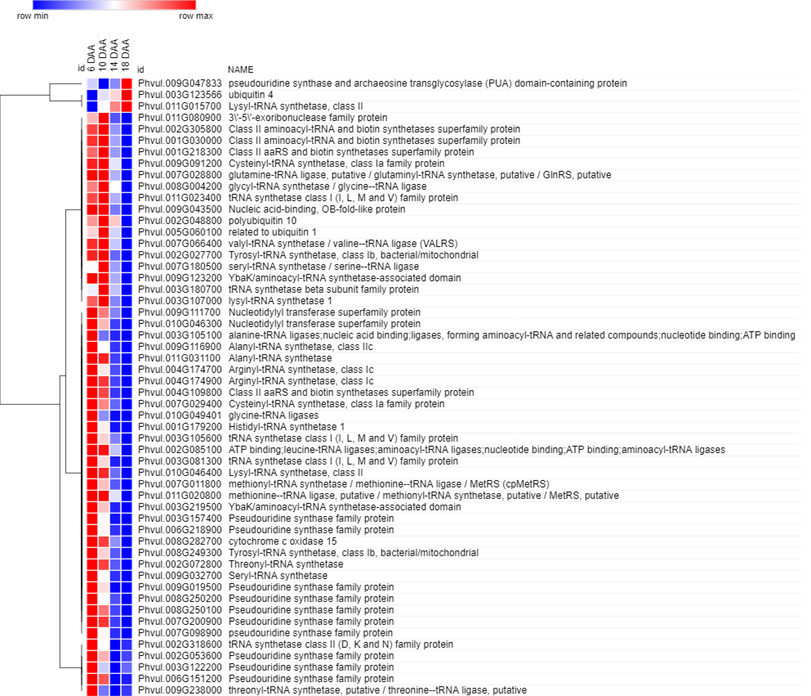
**

**Supplementary Figure 1.** Heatmap of transcripts involved in protein amino acid activation between the time points studied - 6, 10, 14 and 18 days after anthesis (DAA). FPKMs were clustered using Euclidean distance and an average linkage.


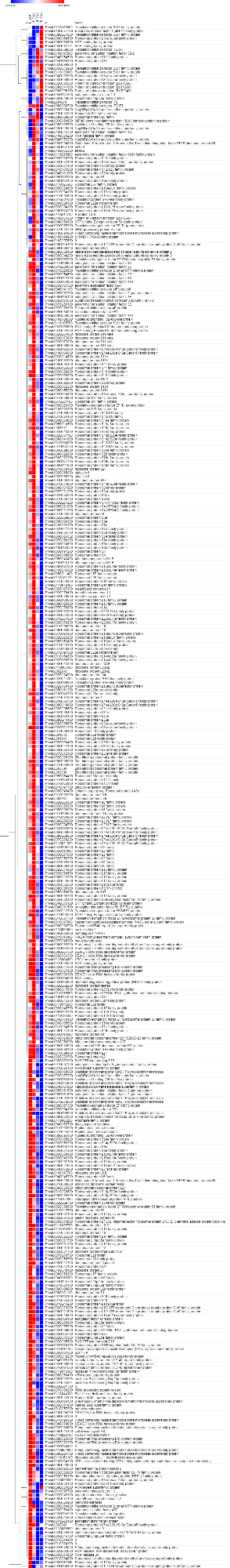


**Supplementary Figure 2.** Heatmap of transcripts involved in protein synthesis between the time points studied - 6, 10, 14 and 18 days after anthesis (DAA). FPKMs were clustered using Euclidean distance and an average linkage.


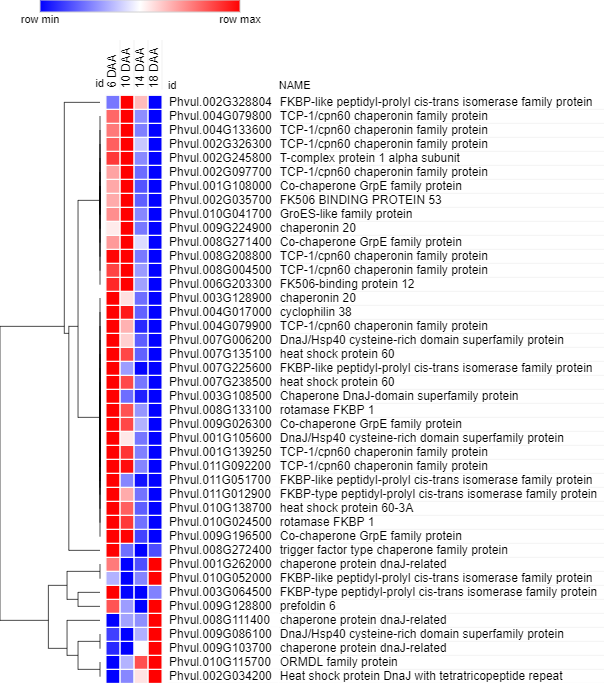


**Supplementary Figure 3.** Heatmap of transcripts involved in protein folding between the time points studied - 6, 10, 14 and 18 days after anthesis (DAA). FPKMs were clustered using Euclidean distance and an average linkage.


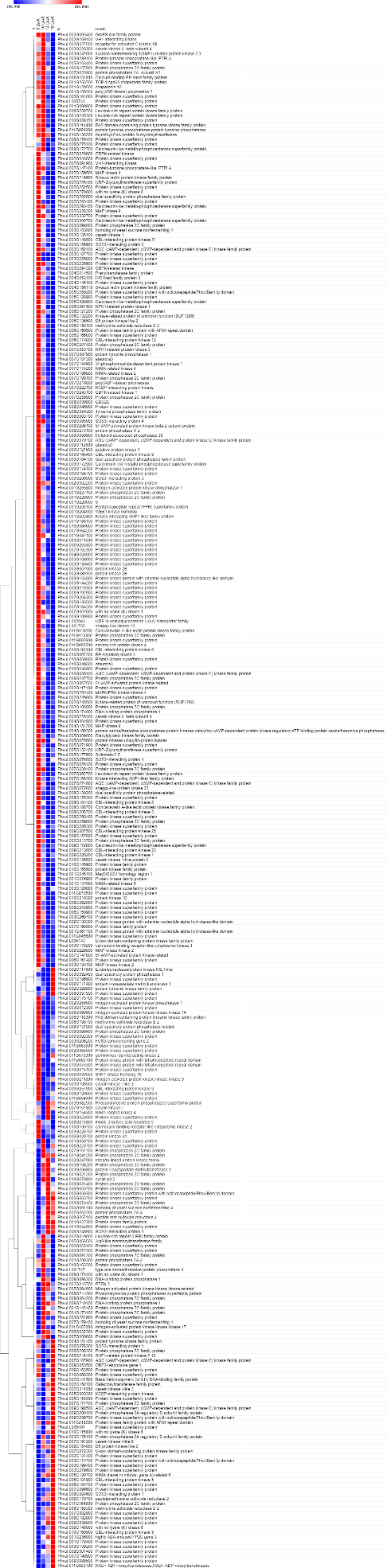


**Supplementary Figure 4.** Heatmap of transcripts involved in protein post-translational modification between the time points studied - 6, 10, 14 and 18 days after anthesis (DAA). FPKMs were clustered using Euclidean distance and an average linkage.


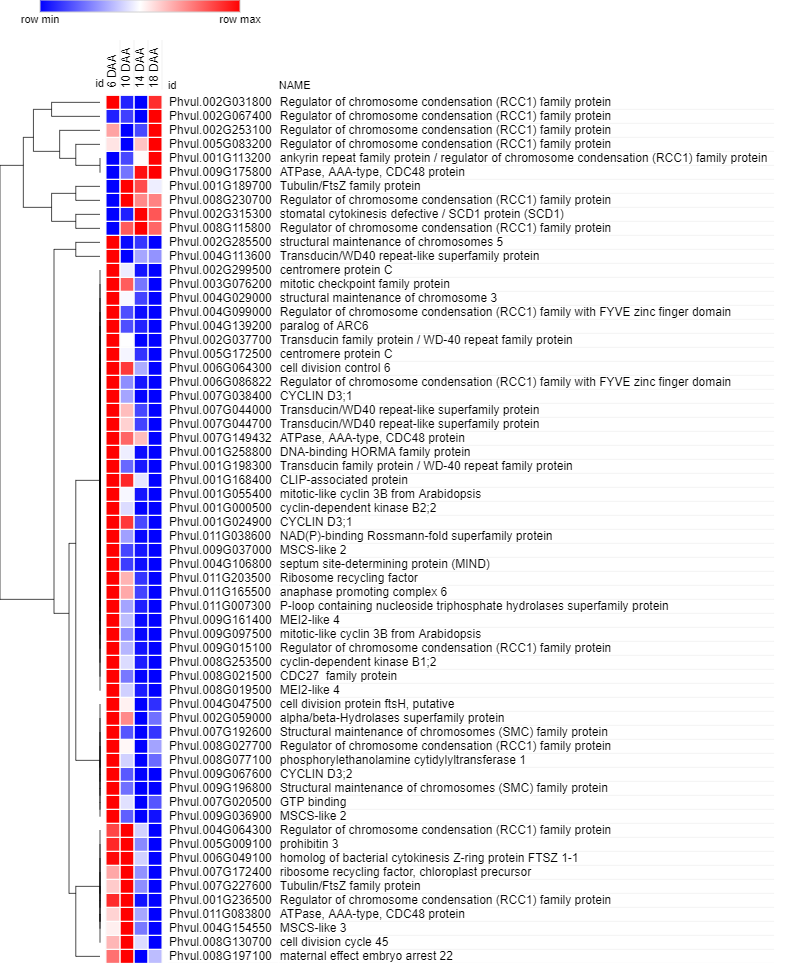


**Supplementary Figure 5.** Heatmap of transcripts involved in cell division between the time points studied - 6, 10, 14 and 18 days after anthesis (DAA). FPKMs were clustered using Euclidean distance and an average linkage.


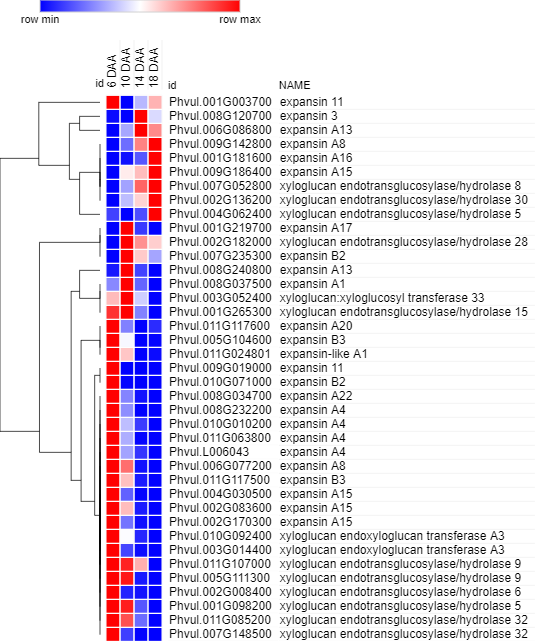


**Supplementary Figure 6.** Heatmap of transcripts involved in cell wall modification (expansion) between the time points studied - 6, 10, 14 and 18 days after anthesis (DAA). FPKMs were clustered using Euclidean distance and an average linkage.


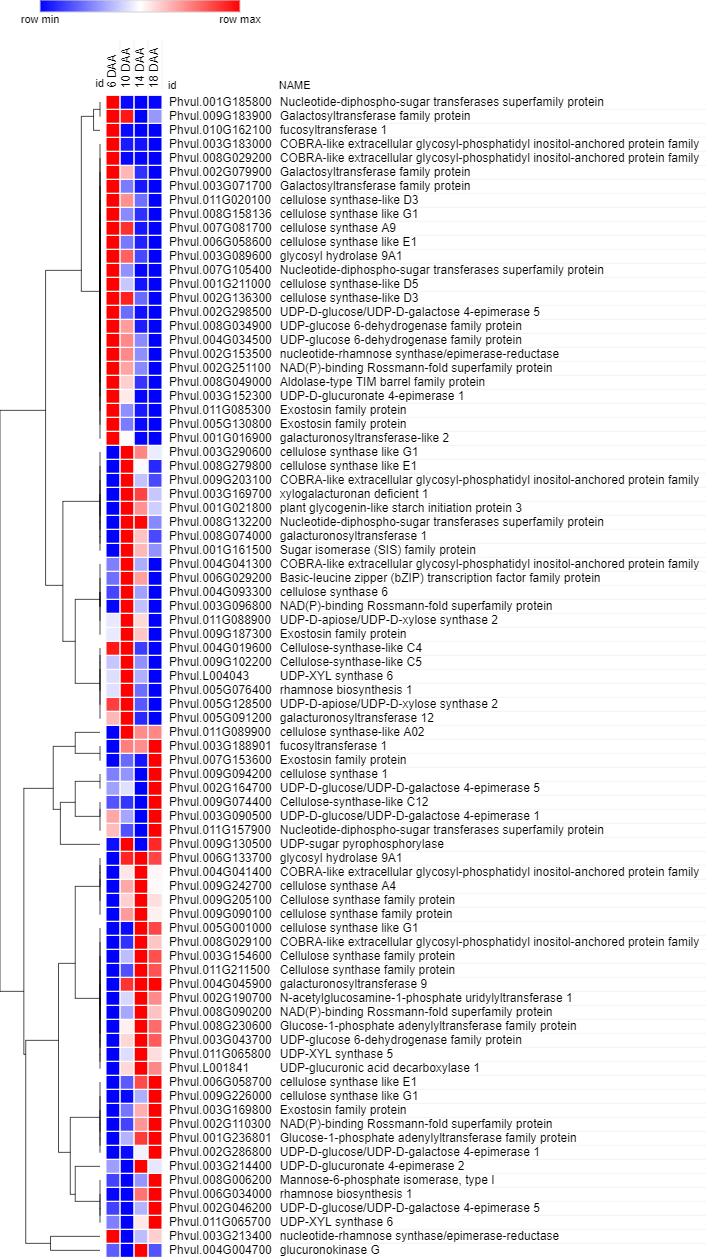


**Supplementary Figure 7.** Heatmap of transcripts involved in cell wall synthesis between the time points studied - 6, 10, 14 and 18 days after anthesis (DAA). FPKMs were clustered using Euclidean distance and an average linkage.


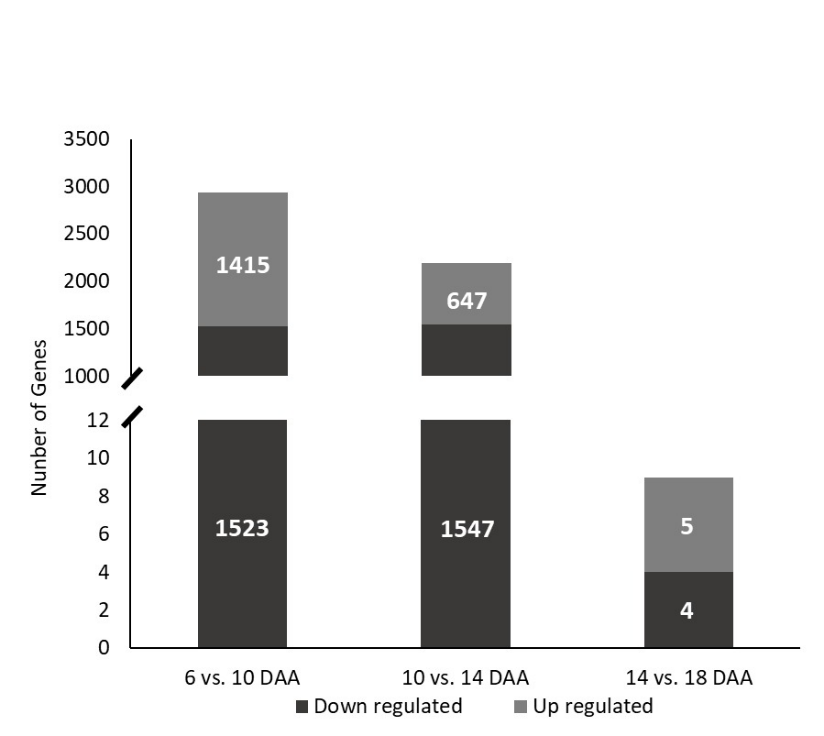


**Supplementary Figure 8.** Number of up- (grey square) and downregulated (black square) genes in the early stages of developing seeds of *P. vulgaris* with a minimum of 100 reads and a Log2FC |≥1| and a adj. p value ≤0.001. DAA: days after anthesis.


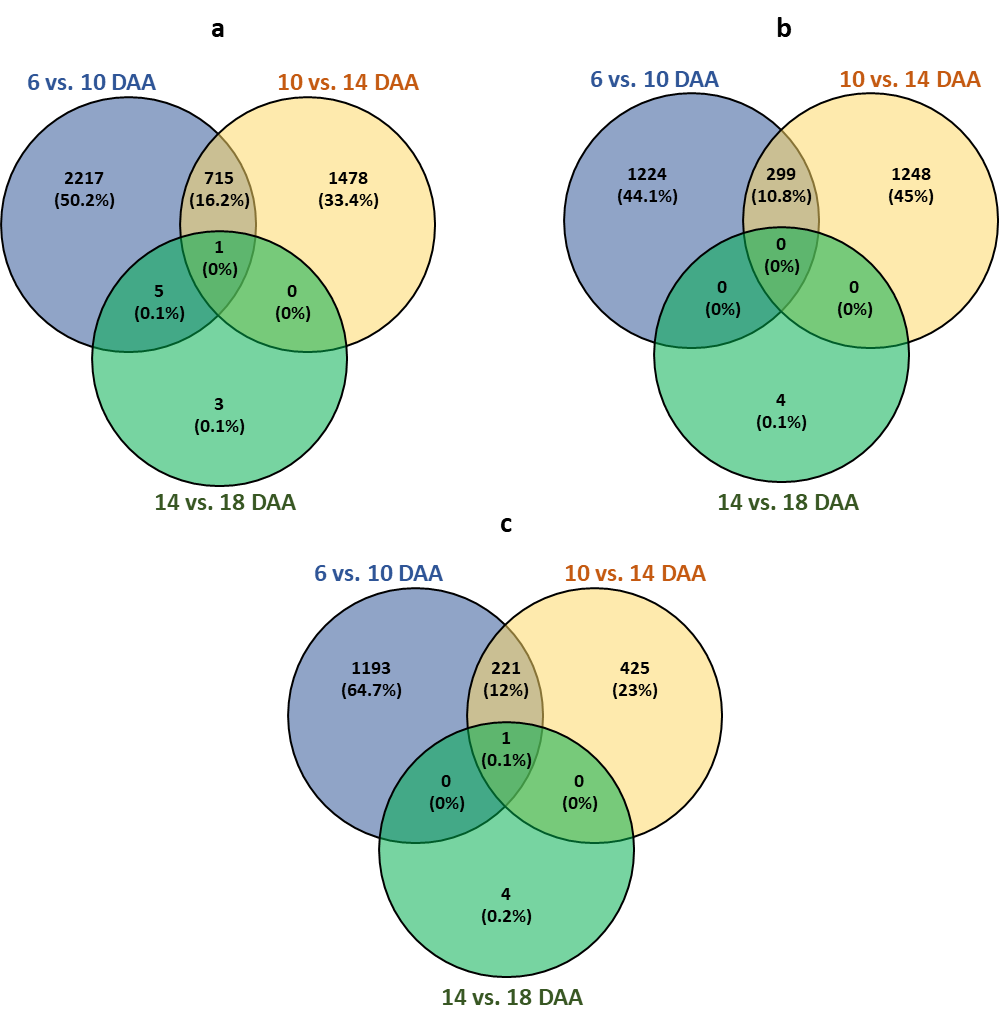


**Supplementary Figure 9.** Venn diagram analysis of differentially expressed genes identified in the transition from the embryo development to seed filling in *P. vulgaris* seeds. The overlapping regions denote common genes among the comparisons studied – 6 vs. 10, 10 vs. 14, 14 vs 18 days after anthesis (DAA). a Total DEGs; b downregulated genes; c upregulated genes.


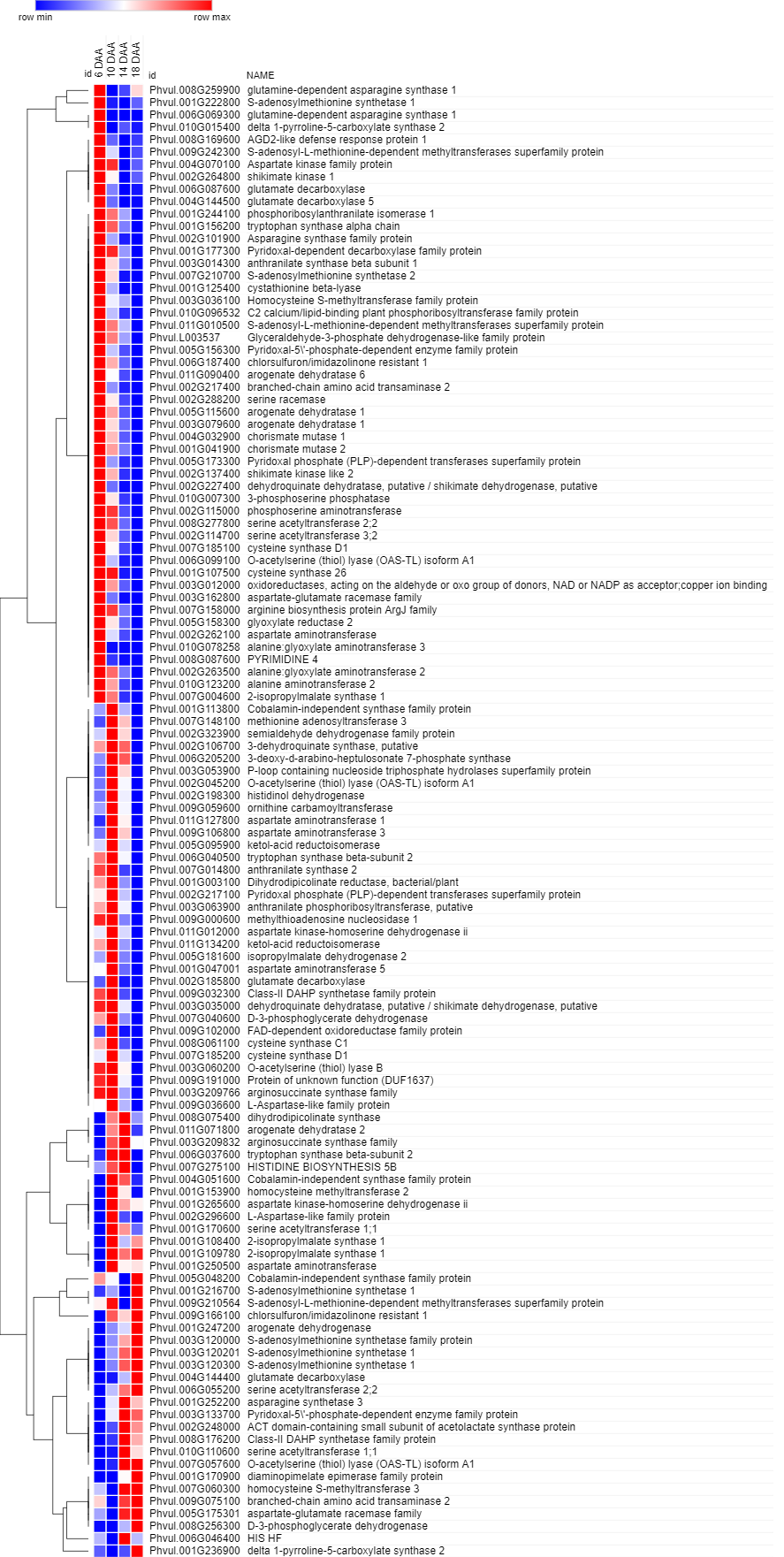


**Supplementary Figure 10.** Heatmap of transcripts involved in amino acid synthesis between the time points studied - 6, 10, 14 and 18 days after anthesis (DAA). FPKMs were clustered using Euclidean distance and an average linkage.


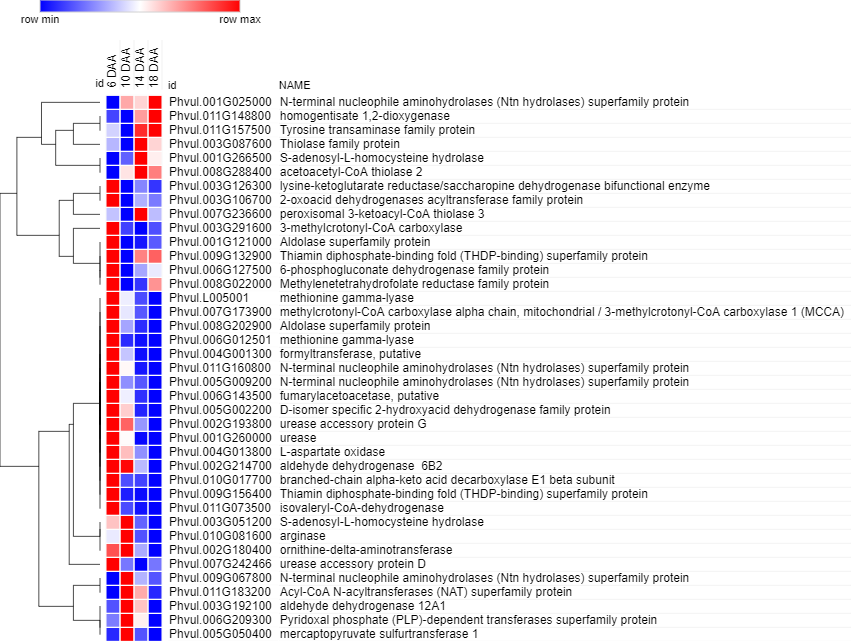


**Supplementary Figure 11.** Heatmap of transcripts involved in amino acid degradation between the time points studied - 6, 10, 14 and 18 days after anthesis (DAA). FPKMs were clustered using Euclidean distance and an average linkage.


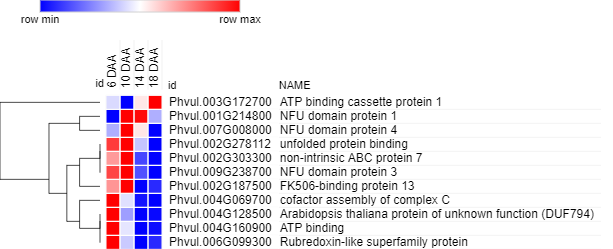


**Supplementary Figure 12.** Heatmap of transcripts involved in protein assembly between the time points studied - 6, 10, 14 and 18 days after anthesis (DAA). FPKMs were clustered using Euclidean distance and an average linkage.

**
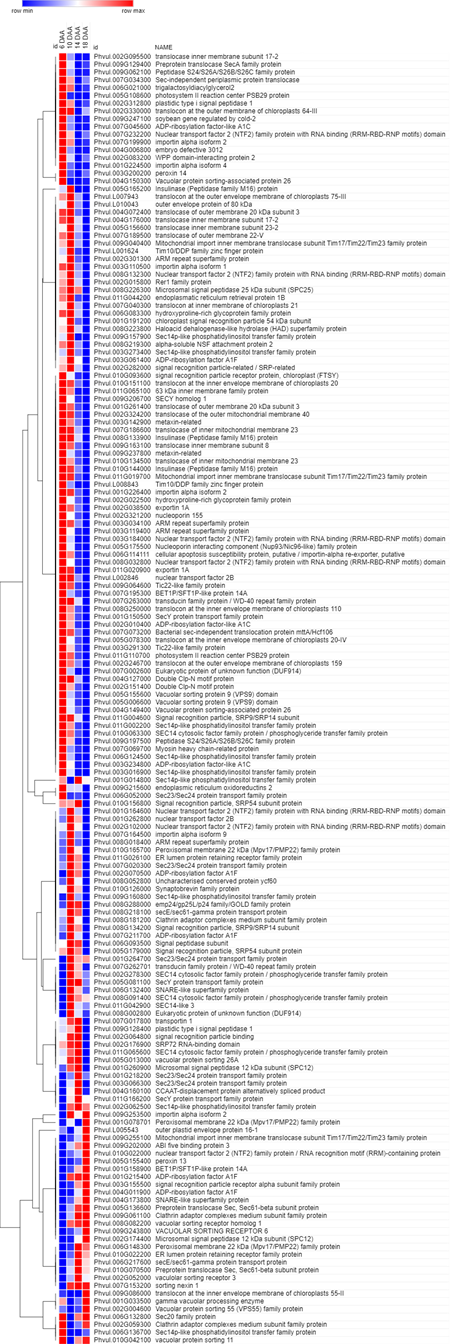
**

**Supplementary Figure 13.** Heatmap of transcripts involved in protein targeting between the time points studied - 6, 10, 14 and 18 days after anthesis (DAA). FPKMs were clustered using Euclidean distance and an average linkage.
